# Genomic prediction of hybrid crops allows disentangling dominance and epistasis

**DOI:** 10.1093/genetics/iyab026

**Published:** 2021-04-14

**Authors:** David González-Diéguez, Andrés Legarra, Alain Charcosset, Laurence Moreau, Christina Lehermeier, Simon Teyssèdre, Zulma G Vitezica

**Affiliations:** 1 INRAE, INP, UMR 1388 GenPhySE, F-31326 Castanet-Tolosan, France; 2 GQE‐Le Moulon, INRAE, Univ. Paris‐Sud, CNRS, AgroParisTech, Université Paris‐Saclay, Gif‐sur‐Yvette, France; 3 Genetics and Analytics Unit, RAGT2n, Druelle, France

**Keywords:** dominance, epistasis, genetic variance, heterosis, genomic models, genomic prediction, GenPred, shared data resources

## Abstract

We revisited, in a genomic context, the theory of hybrid genetic evaluation models of hybrid crosses of pure lines, as the current practice is largely based on infinitesimal model assumptions. Expressions for covariances between hybrids due to additive substitution effects and dominance and epistatic deviations were analytically derived. Using dense markers in a GBLUP analysis, it is possible to split specific combining ability into dominance and across-groups epistatic deviations, and to split general combining ability (GCA) into within-line additive effects and within-line additive by additive (and higher order) epistatic deviations. We analyzed a publicly available maize data set of Dent × Flint hybrids using our new model (called GCA-model) up to additive by additive epistasis. To model higher order interactions within GCAs, we also fitted “residual genetic” line effects. Our new GCA-model was compared with another genomic model which assumes a uniquely defined effect of genes across origins. Most variation in hybrids is accounted by GCA. Variances due to dominance and epistasis have similar magnitudes. Models based on defining effects either differently or identically across heterotic groups resulted in similar predictive abilities for hybrids. The currently used model inflates the estimated additive genetic variance. This is not important for hybrid predictions but has consequences for the breeding scheme—*e.g.* overestimation of the genetic gain within heterotic group. Therefore, we recommend using GCA-model, which is appropriate for genomic prediction and variance component estimation in hybrid crops using genomic data, and whose results can be practically interpreted and used for breeding purposes.

## Introduction

Many plant species are presently cultivated in the form of single-cross hybrid varieties, especially when a strong heterosis effect is observed for yield-related traits (*e.g.* maize, sunflower, sugarbeet, etc.). These hybrids are generally obtained by crossing inbred lines originated from two complementary populations, called heterotic groups. Breeders’ objective is therefore to identify (i) the best single-cross hybrids among all possible crosses between existing inbred lines from the two groups and (ii) create new lines within heterotic group, from crosses of existing lines, which will improve the performance of candidate hybrids at a next generation. Models for genetic improvement of hybrid crops (*e.g.* maize) across two heterotic groups are typically based on the notions of general combining ability (GCA) and specific combining ability (SCA) (Griffing 1962; Stuber and Cockerham 1966; Bernardo 2010). The genotypic value $G_{ij}$ of the cross of lines *i* and *j*, as a function of uniting gametes from *i* and *j*, can be written as follows:

(1) $$G_{ij}=\mu +GCA_{i}+GCA_{j}+SCA_{ij}$$

where GCA of line *i* is the average effect of a gamete when ideally crossed to all gametes from the reciprocal heterotic group. SCA of the combination of line *i* and *j* is the remainder.

It is important to notice, for readers not familiar with hybrid crops, that in many hybrid crops such as maize, parents are pure homozygous individuals (inbred lines). Thus, all gametes produced by *i* (and *j*) are identical, and all F1 descendants of *i* and *j* are identical. This is different from crosses of other species such as animals (pigs for instance) where full-sibs show genetic variation. As a result, GCA contains single locus (additive, in the statistical sense) and multiple loci (additive by additive and higher additive interactions) effects. This is because the whole genotype (gamete) of the pure line is transmitted to the F_1_ descendants, including any possible epistatic combination, and regardless of whether loci in interaction are in the same or in different chromosomes. In this, GCA is different from the concept of Breeding Value in Animal Genetics, which captures the part of functional epistatic effects that is contained in the additive substitution effects, but it does not contain epistatic deviations as they are broken down by meiosis.

Informally, the GCAs within group 1 (group 2) are the sum of additive, additive x additive, additive x additive x additive… deviations *within* group 1 (group 2), whereas SCA are the sum of dominance, all epistatic interaction involving dominance, and any epistatic additive interaction across both groups.

Stuber and Cockerham (1966) presented the covariance across GCAs of different lines and SCAs of different pairs of lines as a function of probabilities of alleles at loci being identical by descent. These probabilities are, in their work, implicitly based on pedigree, and are the same across all loci or pairs of loci. For this reason, and based on pedigree of lines alone, some components of the variance cannot be distinguished; for instance, dominance and across-groups additive epistasis cannot be separated (Bernardo 2010).

With the advent of molecular markers (SNPs), it is possible to obtain better predictions based, ultimately, on similarity across lines (or pairs of lines) measured using markers, using for instance kinship matrices based on VanRaden (2008). Bernardo (1994) transposed the concepts of Stuber and Cockerham (1966) to the prediction of hybrid lines of corn using molecular markers. These ideas have been used extensively (*e.g.*Massman *et al.* 2013; Technow *et al.* 2014; Bouvet *et al.* 2016; Kadam *et al.* 2016; Acosta-Pech *et al.* 2017; Westhues *et al.* 2017; Schrag *et al.* 2018). However, the infinitesimal assumptions of Stuber and Cockerham (1966) are not needed or completely pertinent anymore. SNP markers allow finer distinction of patterns of relationships across and within regions. For instance, it is possible to distinguish relationship within locus and across loci—thus making it possible to split dominance and across-groups epistasis. Also, relationships across dominance deviations are not simple expressions built from additive relationships (Vitezica *et al.* 2013, 2017). Therefore, it is important to properly re-define statistical models for genomic prediction, in order to have models that are more adequate to the physical nature of the genome (finite and not infinitesimal), better understood, and potentially more accurate. The model including a GCA component for each group is convenient because individuals are selected within groups.

In this work we develop a new model, called GCA-model, which corresponds to Stuber and Cockerham (1966) and to Bernardo (2010) ideas, and whose theory we re-develop from scratch for the case of markers. We rederive orthogonal models for genomic prediction in hybrid crops using the notions of effects defined “according to origin” (GCAs and SCAs), using quantitative genetics theory and considering the substitution effects of markers. We present expressions for additive, dominant and epistatic relationships across pure lines and hybrids. Then, as an illustration, we use our method, in an increasing order of complexity, to estimate variance components and predict hybrid performance using a publicly available data set (Technow *et al.* 2014). We compare results of our orthogonal model (GCA-model) with effects defined “according to origin” to the model with effects “defined uniquely” at the hybrid level (G-model), and to the existing formulation of Technow *et al.* (2014) that we show to be a simplification of our approach.

## Theory

Here we derive the theory for genomic relationship matrices in the analysis of hybrid crosses of inbred lines from two populations. We draw on the tradition of separately modeling effects of gametes coming from each heterotic group (Sprague and Tatum 1942; Griffing 1962; Stuber and Cockerham 1966; Bernardo 2010). To derive the genetic model, an ideal (issued from random mating of heterotic pools), and large population of hybrids was assumed. In this way the content of a random sample of gametes depends on the allele frequency in the population. This is the traditional treatment in Quantitative Genetics. The two parental populations or heterotic groups (*e.g.* Dent and Flint in the case of North European maize) are named 1 and 2. Extension to more than two heterotic groups is immediate.

Our aim is to split the total genotypic value of a single-cross hybrid in two statistical additive effects (one from each group), a single dominance deviation (particular to each cross) and epistatic interactions (either intra-group or across-groups). Ideally, this partition is orthogonal and all components should be estimable. Orthogonality is a definitional system that guarantees that variance components are “a priori” (before seeing the data) independent, whereas practical orthogonality depends on the information available in the data set. To our knowledge, this partition and its use in hybrid crops using markers have not been presented elsewhere.

### Additive substitution effects and dominance deviations in hybrid crops

We start from a genotypic model to derive statistical effects (Falconer 1981; Bernardo 2010). Consider a biallelic single locus/gene with alleles *B/b* and the allele origin denoted as *i *=* *1 and *j *=* *2, thus population 1 has $B_{1}$ and $b_{1}$ with respective frequencies $p_{1}\;$and $q_{1}=1-p_{1}$ and population 2 has $B_{2}$ and $b_{2}$ with frequencies $p_{2}$ and $q_{2}$. The hybrid population has genotypes (frequencies) $B_{1}B_{2}$ ($p_{1}p_{2}$), $B_{1}b_{2}\;$($p_{1}q_{2}$), $b_{1}B_{2}\;$($q_{1}p_{2}$) and $b_{1}b_{2}$ ($q_{1}q_{2}$).

We assume additive and dominant gene action, and separate effects of gametes coming from 1 and 2. Then the genotypic value G of a hybrid can be written (up to a common constant) as

$$G_{B_{1}B_{2}}=a_{1}+a_{2}\;G_{B_{1}b_{2}}=a_{1}+d\;G_{b_{1}B_{2}}=a_{2}+d\;G_{b_{1}b_{2}}=0.$$

where $a_{1}$ is the functional additive effect for $B_{1}$ from P1, $a_{2}$ is the functional additive effect for $B_{2}$ from P2, and *d* is the functional dominance value of both heterozygotes ($B_{1}b_{2}$ and $b_{1}B_{2}$). The genetic mean of the hybrid population is therefore

$$E\left(G\right)=p_{1}p_{2}\left(a_{1}+a_{2}\right)+p_{1}q_{2}\left(a_{1}+d\right)+q_{1}p_{2}\left(a_{2}+d\right)=p_{1}a_{1}+p_{2}a_{2}+\left(p_{1}q_{2}+q_{1}p_{2}\right)d$$

Classically, the genotypic values of a hybrid are split into *statistical* additive values, one per parent, and a dominant deviation for the hybrid, as in (Bernardo 2010):

$$G=E\left(G\right)+g_{A^{\left(1\right)}}+g_{A^{\left(2\right)}}+g_{D}$$

where $g_{A^{\left(1\right)}}$ ($g_{A^{\left(2\right)}}$) is the additive effect of a gamete from population 1 (from population 2) combined with a gamete from population 2 (population 1), whereas $g_{D}$ is the dominant deviation.

Additive values $g_{A^{\left(1\right)}}$ and $g_{A^{\left(2\right)}}$ of the gametes include average effects of each gene/allele. The *average effect of alleles* ($\alpha_{B_{1}}$, $\alpha_{b_{1}}$, $\alpha_{B_{2}}$ and $\alpha_{b_{2}}$) are derived from the genotypic values. The reasoning is set out in Table 1 (following Table 7.2 in Falconer (1981)). If gametes carrying $B_{1}$ from population 1 are mated at random with gametes from population 2, the frequencies of the genotypes produced will be $p_{2}$ of $B_{1}B_{2}$ and $q_{2}$ of $B_{1}b_{2}$. The genotypic value of a hybrid $B_{1}B_{2}$ is $G_{B_{1}B_{2}}=(a_{1}+a_{2})$, that of $B_{1}b_{2}$ is $G_{B_{1}b_{2}}=(a_{1}+d)$, and the mean of these two, taking into account of the proportions in which they occur is $E\left(G|B_{1}\right)=p_{2}\left(a_{1}+a_{2}\right)+q_{2}\left(a_{1}+d\right)$.

**Table:** 

Genotype	Frequency	$g_{A^{\left(1\right)}}^{2}$	$g_{A^{\left(1\right)}}$
$B_{1}$	$p_{1}$	${\left(q_{1}\alpha_{1}\right)}^{2}$	$q_{1}\alpha_{1}$
$b_{1}$	$q_{1}$	${\left(-p_{1}\alpha_{1}\right)}^{2}$	${-p}_{1}\alpha_{1}$

**Table 1:** Representation of the average effect of a gene

	Values and frequencies of genotypes produced			
Type of gamete	$B_{1}B_{2}\;{(a}_{1}+a_{2})$	$B_{1}b_{2}\;\left(a_{1}+d\right)$	$b_{1}B_{2}\;\left(a_{2}+d\right)$	$b_{1}{b_{2}}_{\;}\;\left(0\right)$
$B_{1}$	$p_{2}$	$q_{2}$		
$b_{1}$			$p_{2}$	$q_{2}$
$B_{2}$	$p_{1}$		$q_{1}$	
$b_{2}$		$p_{1}$		$q_{1}$

**Table:** 

	Genotype at P2		
Genotype at P1		$B_{2}B_{2}$	$b_{2}b_{2}$
	$B_{1}B_{1}$	$a_{1}+a_{2}$	$a_{1}+d$
	$b_{1}b_{1}$	$a_{2}+d$	0

**Table:** 

	Genotype at P2		
Genotype at P1		$B_{2}B_{2}$	$b_{2}b_{2}$
	$B_{1}B_{1}$	$q_{1}a_{1}+q_{2}a_{2}-\left(p_{1}q_{2}+q_{1}p_{2}\right)d$	$q_{1}a_{1}-p_{2}a_{2}+\left(1-p_{1}q_{2}-q_{1}p_{2}\right)d$

	$b_{1}b_{1}$	$q_{2}a_{2}-p_{1}a_{1}+\left(1-p_{1}q_{2}-q_{1}p_{2}\right)d$	$-p_{1}a_{1}-p_{2}a_{2}-\left(p_{1}q_{2}+q_{1}p_{2}\right)d$

**Table:** 

	Genotype at P2		
Genotype at P1		$B_{2}B_{2}$	$b_{2}b_{2}$
	$B_{1}B_{1}$	$q_{1}\alpha_{1}+q_{2}\alpha_{2}$	$q_{1}\alpha_{1}-p_{2}\alpha_{2}$
	$b_{1}b_{1}$	$-p_{1}\alpha_{1}+q_{2}\alpha_{2}$	$-p_{1}\alpha_{1}-p_{2}\alpha_{2}$

For instance, considering allele $B_{1}$, the difference between the mean value conditional on a particular genotype of the gamete (*e.g.* $E\left(G|B_{1}\right)$) and the population mean $\left(E\left(G\right)\right)$ is the average effect of the allele, $\alpha_{B_{1}}$. The average effects of alleles ($\alpha_{B_{1}}$, $\alpha_{b_{1}}$, $\alpha_{B_{2}}$ and $\alpha_{b_{2}}$) are therefore

$$\alpha_{B_{1}}=p_{2}\left(a_{1}+a_{2}\right)+q_{2}\left(a_{1}+d\right)-E\left(G\right)=q_{1}\left[a_{1}+\left(q_{2}-p_{2}\right)d\right]$$

$$\alpha_{b_{1}}=p_{2}\left(a_{2}+d\right)-E\left(G\right)=-p_{1}\left[a_{1}+\left(q_{2}-p_{2}\right)d\right]$$

$$\alpha_{B_{2}}=p_{1}\left(a_{1}+a_{2}\right)+q_{1}(a_{2}+d)-E(G)=q_{2}\left[a_{2}+\left(q_{1}-p_{1}\right)d\right]$$

$$\alpha_{b_{2}}=p_{1}\left(a_{1}+d\right)-E(G)=-p_{2}\left[a_{2}+\left(q_{1}-p_{1}\right)d\right]$$

Now, we can derive the *average effect of the allele-substitution*, for instance, letting $b_{1}$ be substituted by $B_{1}$. From the $b_{1}$ alleles taken at random from the population for substitution, a proportion $p_{2}$ will be found in $b_{1}B_{2}$ genotypes and a proportion $q_{2}$ in $b_{1}b_{2}$. The substitution will, respectively, change the value from $\left(a_{2}+d\right)$ to $\left(a_{1}+a_{2}\right)$ and from 0 to $\left(a_{1}+d\right)$ (see Table 1). Thus, the average effect of the allele-substitution $\left(\alpha_{1}\right)$ of population 1 is

$$\alpha_{1}=p_{2}\left[\left(a_{1}+a_{2}\right)-\left(a_{2}+d\right)\right]+q_{2}\left[\left(a_{1}+d\right)-0\right]=a_{1}+\left(q_{2}-p_{2}\right)d$$

The same result can be obtained as the difference between average effects: $\alpha_{1}=\alpha_{B_{1}}-\alpha_{b_{1}}$. For population 2 $\left(\alpha_{2}\right)$, it is $\alpha_{2}=\alpha_{B_{2}}-\alpha_{b_{2}}$. The average effects of alleles can also be rewritten as function of the allele substitution effect as follows: $\alpha_{B_{1}}=q_{1}\alpha_{1}$, $\alpha_{b_{1}}=-p_{1}\alpha_{1}$, $\alpha_{B_{2}}=q_{2}\alpha_{2}$ and $\alpha_{b_{2}}=-p_{2}\alpha_{2}$.

Note that allele-substitution effects involve both functional additive ${(a}_{1}+a_{2})$ and dominant (d) effects, and allele frequencies of the other parental population. Similar expressions were presented by Vitezica *et al.* (2016) but an identical functional additive effect ${(a=a}_{1}=a_{2})$ was assumed in both parental populations, ignoring the origin of the allele.

The *statistical additive effects* of a gamete are equal to the sum of the average effects of the alleles it carries. Thus, for a single locus, the statistical additive effects are

$$g_{A_{B_{1}}^{\left(1\right)}}=q_{1}\alpha_{1}$$

$$g_{A_{b_{1}}^{\left(1\right)}}=-p_{1}\alpha_{1}$$

$$g_{A_{B_{2}}^{\left(2\right)}}=q_{2}\alpha_{2}$$

$$g_{A_{b_{2}}^{\left(2\right)}}=-p_{2}\alpha_{2}.$$

This can also be written as $g_{A^{\left(1\right)}}=z_{1}\alpha_{1}$ and $g_{A^{\left(2\right)}}=z_{2}\alpha_{2}$ with z1= 1-p1-p1 for gametes  B1b1, and $z_{2}=\left\{\begin{array}{c} \left(1-p_{2}\right)\\ -p_{2} \end{array}\right.$ for gametes  B2b2

Subtracting statistical additive effects from genotypic values ($G_{B_{1}B_{2}}$, $G_{B_{1}b_{2}}$, $G_{b_{1}B_{2}}$ and $G_{b_{1}b_{2}}$) gives *dominance deviations* which are interactions between the alleles received from parental populations. This is detailed in the Appendix, and the dominance deviation of the hybrid according to its genotype is

$$g_{D_{B_{1}B_{2}}}=-2q_{1}q_{2}d$$

$$g_{D_{B_{1}b_{2}}}=2q_{1}p_{2}d$$

$$g_{D_{b_{1}B_{2}}}=2p_{1}q_{2}d$$

$$g_{D_{b_{1}b_{2}}}=-2p_{1}p_{2}d$$

So, the dominance deviation of a hybrid individual can be written as $g_{D}=wd$ with

$$w=\left\{\begin{array}{c} -2q_{1}q_{2}\\ 2q_{1}p_{2}\\ 2p_{1}q_{2}\\ -2p_{1}p_{2} \end{array}\right.\;\mathrm{for}\;\mathrm{genotypes}\;\left\{\begin{array}{c} B_{1}B_{2}\\ B_{1}b_{2}\\ b_{1}B_{2}\\ b_{1}b_{2} \end{array}\right.$$

Or, equivalently, $w=-2z_{1}z_{2}$ for $z_{1}$, $z_{2}$ defined as above.

Finally, the model for analysis of hybrid crosses considering additive and dominance effects can be written in matrix form for a set of crosses as

$$y=1\mu +g_{A^{\left(1\right)}}+g_{A^{\left(2\right)}}+g_{D}$$

where, for a single locus, $g_{A^{\left(1\right)}}=z_{1}\alpha_{1}$, $g_{A^{\left(2\right)}}=z_{2}\alpha_{2}$, $g_{D}=wd$

### Derivation of additive and dominance genomic relationships

Now we extend the analysis to multiple markers, using $g_{A^{\left(1\right)}}=Z_{1}\alpha_{1}$, $g_{A^{\left(2\right)}}=Z_{2}\alpha_{2}$, $g_{D}=\mathrm{Wd}$, where $Z_{1}=\left(z_{1_{1}}\ldots z_{1_{\mathrm{nsnp}}}\right)$ and $Z_{2}=\left(z_{2_{1}}\ldots z_{2_{\mathrm{nsnp}}}\right)$ are matrices with as many rows as inbred lines present in each heterotic group, and as many columns as the number of markers, nsnp. The matrix $W=\left(w\ldots w_{\mathrm{nsnp}}\right)$ has as many rows as hybrid individuals and as many columns as markers.

Genotypes in pure lines are in matrices $M_{1}\;$and $M_{2}$ which contain zero for genotypes $b_{1}b_{1}$ and $b_{2}b_{2}$, respectively; and 1 for genotypes $B_{1}B_{1}$ and $B_{2}B_{2}$, respectively. The observed *B* allele frequencies for marker *j* in the heterotic groups composed by *n* lines can be computed as $p_{j}=\frac{\sum_{i=1}^{n}M_{ij}}{n}$. Matrices Z are obtained subtracting ***p*** (which is equal to centering if ***p*** is computed from observed genotypes), as $Z_{1}=M_{1}-1p\prime$ for population 1. It is analogous for population 2.

Now, we can set up the covariance matrices.

*Additive covariance matrix* for population 1 (for $g_{A^{\left(1\right)}}$) assuming linkage equilibrium, is

$$\mathrm{Var}\left(g_{A^{\left(1\right)}}\right)=Z_{1}Z_{1}^{\prime }Var\left(\alpha_{1}\right)=Z_{1}Z_{1}^{\prime }\sigma_{\alpha_{1}}^{2}$$

where $\sigma_{\alpha_{1}}^{2}$ is the variance of the allele-substitution effect $\left(\alpha_{1}\right)$ of population 1. For one locus, if the population 1 is a population of pure lines (individuals are homozygotes), the genetic variance *of its gametes* $g_{A^{\left(1\right)}}$ is

$$\sigma_{A^{\left(1\right)}}^{2}=\mathrm{Var}\left(g_{A^{\left(1\right)}}\right)=E\left(g_{A^{\left(1\right)}}^{2}\right)-E{\left(g_{A^{\left(1\right)}}\right)}^{2}$$

By construction of the matrices, $E\left(g_{A^{\left(1\right)}}\right)=0$ and then we have for one locus the following table of gametes and their effects

So, $E(g_{A^{\left(1\right)}}^{2})=p_{1}{\left(q_{1}\alpha_{1}\right)}^{2}+q_{1}{\left(-p_{1}\alpha_{1}\right)}^{2}=p_{1}q_{1}\left(p_{1}+q_{1}\right)\alpha_{1}^{2}=p_{1}q_{1}\alpha_{1}^{2}$ and $\mathrm{Var}\left(g_{A^{\left(1\right)}}\right)=p_{1}q_{1}\alpha_{1}^{2}$.

Assuming linkage equilibrium, we generalize this result to all nsnp markers

$$\sigma_{A^{\left(1\right)}}^{2}=\mathrm{Var}\left(g_{A^{\left(1\right)}}\right)=\sum_{i}^{\mathrm{nsnp}}p_{1_{i}}q_{1_{i}}\;\sigma_{\alpha_{1}}^{2}$$

and

$$\sigma_{\alpha_{1}}^{2}=\frac{\sigma_{A^{\left(1\right)}}^{2}}{\sum_{i}^{\mathrm{nsnp}}p_{1_{i}}q_{1_{i}}\;}$$

Therefore, we can now divide $\mathrm{Var}\left(g_{A^{\left(1\right)}}\right)$ above by this variance and we have

$$\mathrm{Var}\left(g_{A^{\left(1\right)}}\right)=\frac{Z_{1}Z_{1}^{'}}{\sum_{i}^{\mathrm{nsnp}}p_{1_{i}}q_{1_{i}}\;}\sigma_{A^{\left(1\right)}}^{2}=G_{A^{\left(1\right)}}\sigma_{A^{\left(1\right)}}^{2}$$

where $G_{A^{\left(1\right)}}=\frac{Z_{1}Z_{1}^{'}}{\sum_{i}^{\mathrm{nsnp}}p_{1_{i}}q_{1_{i}}\;}$ is the additive genomic relationship matrix across lines in population 1 of size $n_{1}\times n_{1}$. The reasoning is identical for population 2 and only the allele frequencies change.

These results are similar, but not identical, to VanRaden (2008). In particular, using VanRaden’s method 1 directly, while coding genotypes in pure lines as 0/2, results in $G_{VR^{\left(1\right)}\;}=2G_{A^{\left(1\right)}}$. The reason for this discrepancy is because of the reference population used for the additive variance. For a single population with an arbitrary level of inbreeding, the covariance of additive values is expressed as the relationship matrix times the additive variance in an outbred population with the same allele frequencies (Endelman and Jannink 2012). The additive variance, $\sigma_{A^{\left(1\right)}}^{2}$ defined here is for the fully inbred population, and these two definitions of additive variance differ by a factor of 2. VanRaden’s additive relationship matrix divided by two to obtain a kinship (or coancestry) matrix results in $G_{VR^{\left(1\right)}\;}/2$ which is equal to our result. Note that the choice of reference population changes the scaling for G.

For the *dominance deviations*, the covariance matrix for hybrids, assuming linkage equilibrium, is

$$\mathrm{Var}\left(g_{D}\right)=WW^{'}Var\left(d\right)=WW^{'}\sigma_{d}^{2}$$

where $\sigma_{d}^{2}$ is the variance of the dominant effect at the locus level, defined at the hybrid population.

Because gametes are uncorrelated, $\mathrm{Var}\left(g_{D}\right)=\mathrm{Var}\left(wd\right)=\mathrm{Var}\left(-2z_{1}z_{2}d\right)=4d^{2}Var\left(z_{1}z_{2}\right)=4d^{2}Var\left(z_{1}\right)Var\left(z_{2}\right)=4d^{2}p_{1}q_{1}p_{2}q_{2}=4p_{1}q_{1}p_{2}q_{2}d^{2}$. The variance of dominance deviations for F1 hybrids is therefore, for one locus, $\sigma_{D}^{2}=4p_{1}q_{1}p_{2}q_{2}d^{2}$. This is as in Reif *et al.* (2007), Hallauer *et al.* (2010) and Vitezica *et al.* (2016) although the d effect is defined differently in their models, that use “uniquely defined” effects. From here and assuming LE across loci we have

$$\sigma_{D}^{2}=\mathrm{Var}\left(g_{D}\right)=\sum_{i}^{\mathrm{nsnp}}\left(4p_{1_{i}}q_{1_{i}}p_{2_{i}}q_{2_{i}}\right)\;\sigma_{d}^{2}$$

which results in the covariance matrix

$$\mathrm{Var}\left(g_{D}\right)=\frac{WW^{'}}{\sum_{i}^{\mathrm{nsnp}}(4p_{1_{i}}q_{1_{i}}p_{2_{i}}q_{2_{i}})\;}\sigma_{D}^{2}=D\sigma_{D}^{2}$$

where $D=\frac{WW^{'}}{\sum_{i}^{\mathrm{nsnp}}(4p_{1_{i}}q_{1_{i}}p_{2_{i}}q_{2_{i}})\;}$ is the dominance relationship matrix across hybrids of size n×n. Note that we assume that allele substitution effects and dominance effects are random with respective variances $\sigma_{\alpha }^{2}$ and $\sigma_{d}^{2}$.

Technow *et al.* (2014) modelled specific combining abilities using element-by-element products of matrices $G_{A^{\left(1\right)}}$ and $G_{A^{\left(2\right)}}$, following Stuber and Cockerham (1966). Clearly this is not the same as our matrix D above, that results directly from modelling dominance deviations. We will show later that Technow *et al.* (2014) approach, in fact, only models additive by additive across-heterotic groups epistasis, which is indeed a part of the SCA, and that their approach is an approximation to our D. Also we show that the method of Stuber and Cockerham (1966) using element-by-element products to obtain relationships across SCA assumes an infinitesimal model, and should not be directly transposed to marker-based models.

#### Some properties of the additive and dominance relationship matrices

Matrices $G_{A^{\left(1\right)}}$, $G_{A^{\left(2\right)}}$ and D have an average diagonal equal to 1 and an average value equal to 0 across the whole matrix. This implies that estimates of variance components can be interpreted as genetic variances (Legarra 2016). Also, z and w, the underlying incidence matrices to $G_{A^{\left(1\right)}}$, $G_{A^{\left(2\right)}}$ and D are orthogonal (see Appendix for the proof), which implies that by construction statistical estimates are *a priori* independent from each other. Also, because the basic bricks z and w are orthogonal, extension to higher order of interaction (epistasis) is immediate and also orthogonal (as mentioned later). In the next section, we present epistatic relationship matrices.

### Epistasis in hybrid populations

So far, we have written down the model for analysis of hybrid crosses including additive and dominance relationships. Now we use Kronecker products to extend the incidence matrices z and w to epistatic interactions.

The classical model (1) including GCA and SCA effects for a hybrid individual can be written as $y_{ij}=\mu +{\mathrm{gca}}_{i}+{\mathrm{gca}}_{j}+{\mathrm{sca}}_{ij}+e_{ij}$, where $y_{ij}$ is the phenotypic value of the hybrid, μ is the population mean, ${\mathrm{gca}}_{i}$ is the GCA of line i, ${\mathrm{gca}}_{j}$ is the GCA of line j and ${\mathrm{sca}}_{ij}$ is the SCA which depends of the combination of alleles received from *i* and *j*.

#### Epistasis intrapopulation

Stuber and Cockerham (1966) showed that the GCA-term $\left({\mathrm{gca}}_{i}\right)$ includes, in addition to the additive gametic effects, the additive-by-additive epistasis across loci for alleles present in the line (equation 1, page 1279), and all higher order additive interactions in the line. This is because the lines are inbred—so exactly the same gamete is always transmitted to the hybrid, contrary to animal breeding where recombination breaks down epistatic combinations. So, for instance the GCA of a gamete from population 1, considering two loci, *k* and *m* and second-order epistasis,

$$gca_{i}=z_{1\left(k\right)}\alpha_{1}^{k}+z_{1\left(m\right)}\alpha_{1}^{m}+z_{1\left(k\right)}⨂z_{1\left(m\right)}{\left(\alpha \alpha \right)}_{1}^{k,m}$$

where ${\left(\alpha \alpha \right)}_{1}^{k,m}$ is the deviation due to epistatic interaction across loci in population 1. The coefficients of the incidence matrix, $z_{1\left(k\right)}⨂z_{1\left(m\right)}$, for second-order epistatic effects between two loci can be computed as the Kronecker products (⨂) of the respective incidence matrices for single locus effects. A Kronecker product of orthogonal incidence matrices results in an orthogonal incidence matrix (Van Loan 2000), so the orthogonality (always under the assumption of linkage equilibrium) extends to any order of epistasis. For multiple loci, the matrix $Z_{11}$ of additive-by-additive interaction effects can be written using Kronecker products of each row (corresponding to each line) of the preceding matrices as

$$Z_{11}=\left(\begin{array}{c} z_{1_{1}}⨂\;z_{1_{1}}\\ \ldots \\ z_{1_{i}}⨂\;z_{1_{i}}\\ \ldots \\ z_{1_{n_{1}}}⨂\;z_{1_{n_{1}}} \end{array}\right)$$

Matrix $Z_{11}$ is of large size (the number of rows is $\mathrm{nsn}p^{2}$) but it is not explicitely used. Following Vitezica *et al.* (2017), we know that $Z_{11}Z_{11}^{'}=Z_{1}Z_{1}^{'}⊙Z_{1}Z_{1}^{'}$ where ⊙ is the Hadamard product; following developments in Vitezica *et al.* (2017) the genomic additive-by-additive epistatic relationship matrices of lines of population 1 with themselves is thus

$$G_{AA^{\left(1,1\right)}}=\frac{\left(Z_{1}Z_{1}^{'}⊙Z_{1}Z_{1}^{'}\right)}{tr\left(Z_{1}Z_{1}^{'}⊙Z_{1}Z_{1}^{'}\right)/n_{1}}=\frac{G_{A^{\left(1\right)}}⊙G_{A^{\left(1\right)}}}{tr\left(G_{A^{\left(1\right)}}⊙G_{A^{\left(1\right)}}\right)/n_{1}}$$

in agreement (up to a scaling factor) with Cockerham (1954), Henderson (1984), Martini *et al.* (2016), and Vitezica *et al.* (2017).

In the above expression, tr is the trace and $n_{1}$ is the number of lines in population 1. Therefore, the covariance matrix for the additive-by-additive interaction within population 1 $\left(g_{AA^{\left(11\right)}}\right)$ is:

$$\mathrm{Var}\left(g_{AA^{\left(1,1\right)}}\right)=G_{AA^{\left(1,1\right)}}\sigma_{AA^{\left(1,1\right)}}^{2}$$

The reasoning for population 2 is the same resulting in

$$G_{AA^{\left(2,2\right)}}=\frac{\left(Z_{2}Z_{2}^{'}⊙Z_{2}Z_{2}^{'}\right)}{tr\left(Z_{2}Z_{2}^{'}⊙Z_{2}Z_{2}^{'}\right)/n_{2}}=\frac{G_{A^{\left(2\right)}}⊙G_{A^{\left(2\right)}}}{tr\left(G_{A^{\left(2\right)}}⊙G_{A^{\left(2\right)}}\right)/n_{2}}$$

and $\mathrm{Var}\left(g_{AA^{\left(2,2\right)}}\right)=G_{AA^{\left(2,2\right)}}\sigma_{AA^{\left(2,2\right)}}^{2}$

The dimensions of $G_{AA^{\left(2,2\right)}}$ and $G_{AA^{\left(2,2\right)}}$ are $n_{1}\times n_{1}$ and $n_{2}\times n_{2}$, respectively.

#### Epistasis across populations

According to Stuber and Cockerham (1966), the SCA-term $\left({\mathrm{sca}}_{ij}\right),$ in addition to the dominant deviation effects, includes the additive-by-additive epistasis across loci in alleles coming from different populations (equation 2, page 1279), the additive-by-dominant and dominant-by-dominant interactions, plus higher order interactions that we will not detail here as the reasoning is the same. So, SCA for a hybrid from populations 1 and 2, considering two loci, *k* and *m*,

$$sca_{ij}=w_{\left(k\right)}d^{k}+w_{\left(m\right)}d^{m}+z_{1(k)}⨂z_{2\left(m\right)}{\left(\alpha \alpha \right)}_{1,2}^{k,m}+z_{1\left(k\right)}⨂w_{\left(m\right)}{\left(\alpha d\right)}_{1,2}^{k,m}+w_{\left(k\right)}⨂z_{2\left(m\right)}{\left(\alpha d\right)}_{1,2}^{m,k}+w_{\left(k\right)}⨂w_{\left(m\right)}{\left(dd\right)}_{1,2}^{k,m}$$

The different $z_{1}$ and $z_{2}$ come from two parental lines *i* and *j*. Let $T_{1}$ be a matrix relating hybrids to lines in population 1 with 1 in the *k, l* position if the *k-*th hybrid comes from the *l-*th line in population 1 and $T_{2}$ a similar matrix linking hybrids to lines in population 2. The covariance matrix for the additive-by-additive interaction between populations 1 and 2 ($g_{AA^{\left(12\right)}}$) can be calculated as:

$$G_{AA^{\left(1,2\right)}}=\frac{T_{1}G_{A^{\left(1\right)}}T_{1}^{'}⊙T_{2}G_{A^{\left(2\right)}}T_{2}^{'}}{tr\left(T_{1}G_{A^{\left(1\right)}}T_{1}^{'}⊙T_{2}G_{A^{\left(2\right)}}T_{2}^{'}\right)/n\;}$$

$$\mathrm{Var}\left(g_{AA^{\left(12\right)}}\right)=G_{AA^{\left(1,2\right)}}\sigma_{AA^{\left(1,2\right)}}^{2}$$

where n is the number of hybrids and the matrix $G_{AA^{\left(1,2\right)}}$ has size n×n. In other words, the matrix $G_{AA^{\left(1,2\right)}}$ is formed as follows:

1. For each pair of hybrids i,j with respective parents parent1(i) (from population 1), parent2(i) (from population 2) and parent1(j) (from population 1) and parent2(j) (from population 2) do:
  $$G_{AA^{\left(1,2\right)}}\left[i,j\right]=G_{A^{\left(1\right)}}\left[\mathrm{parent}1\left(i\right),\mathrm{parent}1\left(j\right)\right]\times G_{A^{\left(2\right)}}\left[\mathrm{parent}2\left(i\right),\mathrm{parent}2\left(j\right)\right]$$
2. Scale the resulting matrix to an average diagonal of 1.

Technow *et al.* (2014) used $G_{AA^{\left(1,2\right)}}$ to model the relationship matrix of SCA’s. We have shown that this is incorrect because $G_{AA^{\left(1,2\right)}}$ models across-population epistasis (interactions across loci for alleles coming from different heterotic groups) but it does not model dominance deviations (interactions within loci). We will see in the Discussion section that $G_{AA^{\left(1,2\right)}}$ is in fact an *approximation* of D.

Relationships for the other pairwise epistatic interactions (all of them present in the SCA and of size n×n) are:

- Additive in population 1 by dominant: $G_{A^{\left(1\right)}D}=\frac{T_{1}G_{A^{\left(1\right)}}T_{1}^{'}⊙D}{tr\left(T_{1}G_{A^{\left(1\right)}}T_{1}^{'}⊙D\right)/n\;}$
- Additive in population 2 by dominant: $G_{A^{\left(2\right)}D}=\frac{T_{2}G_{A^{\left(2\right)}}T_{2}^{'}⊙D}{tr\left(T_{2}G_{A^{\left(2\right)}}T_{2}^{'}⊙D\right)/n\;}$
- Dominant by dominant: $G_{DD}=\frac{D⊙D}{tr\left(D⊙D\right)/n\;}$

In the same manner, it is possible to derive relationships for third and higher order interactions, using Hadamard products of $G_{A^{\left(1\right)}}$, $G_{A^{\left(2\right)}}$ and

D including the incidence matrices T for across-population interactions.

As the two gametes in each hybrid are uncorrelated, the genotypic variance (Stuber and Cockerham^1966^) (ignoring third and higher order epistatic terms) is

$$\sigma_{G}^{2}=\sigma_{A^{\left(1\right)}}^{2}+\sigma_{A^{\left(2\right)}}^{2}+\sigma_{D}^{2}+\sigma_{AA^{\left(1,1\right)}}^{2}+\sigma_{AA^{\left(2,2\right)}}^{2}+\sigma_{AA^{\left(1,2\right)}}^{2}+\sigma_{A^{\left(1\right)}D}^{2}+\sigma_{A^{\left(2\right)}D}^{2}+\sigma_{DD}^{2}$$

where

$$\sigma_{GCA^{\left(1\right)}}^{2}=\sigma_{A^{\left(1\right)}}^{2}+\sigma_{AA^{\left(1,1\right)}}^{2,},\;\sigma_{GCA^{\left(2\right)}}^{2}=\sigma_{A^{\left(2\right)}}^{2}+\sigma_{AA^{\left(2,2\right)}}^{2}$$

and

$$\sigma_{\mathrm{SCA}}^{2}=\sigma_{D}^{2}+\;\sigma_{AA^{\left(1,2\right)}}^{2}+\sigma_{A^{\left(1\right)}D}^{2}+\sigma_{A^{\left(2\right)}D}^{2}+\sigma_{DD}^{2}.$$

In our analysis on real data, we will ignore the epistatic interaction terms $\sigma_{A^{\left(1\right)}D}^{2},\;\sigma_{A^{\left(2\right)}D}^{2}$ and $\sigma_{DD}^{2}$ as their estimate is very inaccurate.

We remark that a breeder is interested in $\sigma_{\mathrm{GCA}}^{2}$ (because it indicates how much variation is expected in hybrids) but also in $\sigma_{A}^{2}$, which determines the genetic progress of hybrids that is achievable by selecting lines, crossing them, and producing new inbred lines *within* heterotic groups (Stuber and Cockerham 1966). This is because epistasis combinations are broken down by recombination when creating new source populations for line development within each heterotic group.

## Materials and methods

As illustration of the genomic relationship matrices developed here, variance components were estimated using the publicly available data set from the breeding program of the University of Hohenheim (https://doi.org/10.1534/genetics.114.165860) (Technow *et al.* 2014).

### Phenotypes and genotypes

Here, a brief description of the phenotypic and genotypic data set is given (more details in Technow *et al.* (2014)). We analyzed the adjusted entry means of n=1254 single-cross hybrids for grain yield expressed in quintals per hectare (q ha^−1^), representing an incomplete factorial design between $n_{1}=123$ Dent and $n_{2}=86$ Flint inbred lines (two genetically divergent heterotic groups) with high linkage disequilibrium (LD) within heterotic groups. The hybrid data were collected in 14 years (1999-2012) and on average, 95 hybrids produced from 15 Dent and 11 Flint lines, were tested each year.

All parental inbred lines were genotyped with the Illumina Maize SNP50 BeadChip (Ganal *et al.* 2011). Here, we used the 35,478 SNP available after quality control (see details in Technow *et al.* 2014). Markers that were monomorphic in one group but segregating in the other group were kept. Genotypes of tested single-cross hybrids were derived from parental genotypes.

### Genomic evaluation models

Our new GCA-model was compared with Stuber and Cockerham (1966) model for effects of genes “defined uniquely”, whose implementation in a marker based-model for hybrid crops is actually the NOIA system (Álvarez-Castro and Carlborg 2007; Vitezica *et al.* 2017). We will call this the G-model. Both genomic models were used for analysis of hybrid records (either estimation of genetic parameters or cross-validation). Note that the difference from our GCA-model to previous studies is that we use genomic relationship matrices that we have completely developed from theory (and not by transposition of pedigree-based concepts).

*GCA model.* Here, effects were defined “according to origin” (Sprague and Tatum 1942; Griffing 1962; Stuber and Cockerham 1966), but with relationship matrices developed in the Theory section. So, GCA-model for the (i,j) hybrid resulting from the combination of parental lines i (from population 1) and j (from population 2) can be written as:

$$y_{ij}=\mu +gca_{i}^{\left(1\right)}+gca_{j}^{\left(2\right)}+sca_{ij}+e_{ij}$$

where $y_{ij}$ is the hybrid phenotype (entry mean), μ is the overall mean. Our models differ in the explicit modelling of the GCA and SCA into sub-components due to additive, dominant and epistatic statistical effects. In classical settings, GCA and SCA may be modelled either as fixed or as independent random effects (Bernardo 2010; Hallauer *et al.* 2010). For instance, Giraud *et al.* (2017) used a model with random, uncorrelated GCA and SCA for the analysis of a half-diallel design. As mentioned before, GCA contains additive, additive x additive and further within-heterotic epistatic effects, whereas SCA can be split into dominance, across-population additive effects, and epistatic effects including dominance. For instance, $gca^{\left(1\right)}=g_{A^{\left(1\right)}}+g_{AA^{\left(1,1\right)}}+g_{AAA^{\left(1,1\right)}}+g_{\mathrm{AAA}A^{\left(1,1\right)}}+\cdots$ where all terms are obviously not always fit in the model or estimable in practice.

Still, and because there are potentially several hybrids for each line, we use “residual genetic” r effects (*e.g.*Endelman *et al.* 2018) (see explanation below—similar to the “permanent environmental effect” in animal breeding) as a catch-all term that includes genetic effects that are not explicitely included. For instance, if we assume $gca^{\left(1\right)}=g_{A^{\left(1\right)}}+r$, the term r captures $r=g_{AA^{\left(1,1\right)}}+g_{AAA^{\left(1,1\right)}}+g_{\mathrm{AAA}A^{\left(1,1\right)}}\ldots$ and further terms, but if we assume $gca^{\left(1\right)}=g_{A^{\left(1\right)}}+g_{AA^{\left(1,1\right)}}+r$, then $r=g_{AAA^{\left(1,1\right)}}+g_{\mathrm{AAA}A^{\left(1,1\right)}}\ldots$.

The “residual genetic” *r* effects are assumed random and uncorrelated, and can be estimated because there are repeated hybrids for each line. In this manner the models are more robust to the fact of fitting genetic effects up to an arbitrary complexity that may be enough or not. The strategy of fitting “catch-all” “residual genetic” effects could also be followed for the SCA, but because hybrids are not repeated in the entry means, “residual genetic” *r* effects are not estimable as they are confounded with the residual.

Then we make several choices of genetic effects to be explicitly included in GCA and SCA, leading to several models that will be described later. The most complete model includes $gca^{\left(1\right)}=g_{A^{\left(1\right)}}+g_{AA^{\left(1,1\right)}}+r^{\left(1\right)}$, $gca^{\left(2\right)}=g_{A^{\left(2\right)}}+g_{AA^{\left(2,2\right)}}+r^{\left(2\right)}$, $\mathrm{sca}=g_{D}+g_{AA^{\left(1,2\right)}}$. We ignore second-order epistasis including dominance and higher order epistatic interactions. Thus, the most complete model is:

$$y_{ij}=\mu +g_{A^{\left(1\right)}\;i}+g_{A^{\left(2\right)}j}+g_{\mathrm{Dij}}+g_{AA^{\left(1,1\right)}\;i}+g_{AA^{\left(2,2\right)}\;j}+g_{AA^{\left(1,2\right)}\;ij}+r_{i}^{\left(1\right)}+r_{j}^{\left(2\right)}+e_{ij}$$

which in vectorial form (all phenotypes in vector y) is:

$$y=1\mu +T_{1}g_{A^{\left(1\right)}}+T_{2}g_{A^{\left(2\right)}}+g_{D}+T_{1}g_{AA^{\left(1,1\right)}}+T_{2}g_{AA^{\left(2,2\right)}}+g_{AA^{\left(1,2\right)}}+{T_{1}r}^{\left(1\right)}+T_{2}r^{\left(2\right)}+e$$

where the T incidence matrices assign hybrids to parents in each heterotic group.

The additive effects of gametes from each inbred line are assumed distributed as $g_{A^{\left(1\right)}}∼\mathrm{MVN}\left(0,\;G_{A^{\left(1\right)}}\sigma_{A^{\left(1\right)}}^{2}\right)$ and $g_{A^{\left(2\right)}}∼\mathrm{MVN}\left(0,\;G_{A^{\left(2\right)}}\sigma_{A^{\left(2\right)}}^{2}\right)$, the dominance deviation effects for each hybrid combination, as $g_{D}∼\mathrm{MVN}\left(0,\;D\sigma_{D}^{2}\right)$; the epistatic interaction effects within each heterotic group are $g_{AA^{\left(1,1\right)}}∼\mathrm{MVN}\left(0,\;G_{{AA}^{\left(1,1\right)}}\sigma_{{AA}^{\left(1,1\right)}}^{2}\right)$, $g_{AA^{\left(2,2\right)}}∼\mathrm{MVN}\left(0,\;G_{{AA}^{\left(2,2\right)}}\sigma_{{AA}^{\left(2,2\right)}}^{2}\right)$ and between heterotic groups is $g_{AA^{\left(1,2\right)}}∼\mathrm{MVN}\left(0,\;G_{{AA}^{\left(1,2\right)}}\sigma_{{AA}^{\left(1,2\right)}}^{2}\right)$. Finally, $r^{\left(1\right)}$ is the vector of random residual genetic effects $r^{\left(1\right)}∼\mathrm{MVN}\left(0,\;I\sigma_{r^{\left(1,1\right)}}^{2}\right)$, $r^{\left(2\right)}$ is the vector of random residual genetic effects $r^{\left(2\right)}∼\mathrm{MVN}\left(0,\;I\sigma_{r^{\left(2,2\right)}}^{2}\right)$, and e is the vector of random residual effects $e∼\mathrm{MVN}\left(0,\;I\sigma_{e}^{2}\right)$. All the relationship matrices have been defined in the Theory section.

*Fit of “residual genetic” GCA effects.* As discussed before, the GCA effect conceptually contains within-population additive effects and additive epistatic interactions of any order. As not all these interactions are explicitely modelled, and because pure lines are repeated in hybrids, we fit “residual genetic” r effects in GCA-models. For instance, if only additive effects are fit, this “residual genetic” r effect account for all additive epistasis within-group present in the GCA effect. Fitting residual genetic GCA effects is similar to fitting individual permanent environmental effects in animal breeding (*e.g.* for a cow that gives repeated performances of milk yield). It is known in animal breeding that this effect captures, among other things, genetic effects not explicitly modelled such as dominance or epistasis (Kruuk 2004; Vitezica *et al.* 2018). Indeed, historically GCAs have been estimated as random, unrelated effects, for instance in diallel designs (Sprague and Tatum 1942; Hallauer *et al.* 1988). Thus, all the models detailed above (shown in Table 2) include “residual genetic” r effects.

*G model.* This model ignores the origin of the gametes and uses a “uniquely defined” effect per hybrid (Stuber and Cockerham 1966), as developed in a genomic context by Vitezica *et al.* (2017) using the NOIA approach, to correctly model dominance deviations under the constraint that hybrids are not in HWE. The G-model for single-cross hybrid individuals can be written as:

$$y=1\mu +g_{A^{\left(H\right)}}+g_{D^{\left(H\right)}}+g_{{AA}^{\left(H\right)}}+e$$

where $g_{A^{\left(H\right)}}$ are the additive genetic effects of hybrids distributed as $g_{A^{\left(H\right)}}∼\mathrm{MVN}(0,\;G_{A^{\left(H\right)}}\sigma_{A^{\left(H\right)}}^{2})$, the dominant genetic effects are $g_{D^{\left(H\right)}}∼\mathrm{MVN}\left(O,\;D_{H}\sigma_{D^{\left(H\right)}}^{2}\right)$, and the additive-by-additive epistatic interaction effects are $g_{{AA}^{\left(H\right)}}∼\mathrm{MVN}(O,\;G_{AA^{\left(H\right)}}\sigma_{AA^{\left(H\right)}}^{2})$.

The matrices $G_{A^{\left(H\right)}}$, $D_{H}$ and $G_{AA^{\left(H\right)}}$ are the additive, dominant and additive-by-additive genomic relationship matrices, defined as (Vitezica *et al.* 2017)

$$G_{A^{\left(H\right)}}=\frac{H_{a}H_{a}^{'}}{tr(H_{a}H_{a}^{'})/n}$$

where the matrix $H_{a}$ has elements equal to $\left(2\;-\;2p_{k}\right),\;\left(1\;-\;2p_{k}\right),\;-2p_{k}$ for genotypes BB, Bb and bb, and $p_{k}$ is the frequency of B at the $k^{th}$ marker of the hybrid population. It is the same as VanRaden’s **G** but with a different denominator to account for lack of HWE. The dominance matrix $D_{H}$ is

$$D_{H}=\frac{H_{d}H_{d}^{'}}{tr(H_{d}H_{d}^{'})/n}$$

where $H_{d}$ contains elements $h_{d}$ for each individual and locus equal to

$$h_{d}=\{\begin{array}{c} {-2[p_{BB}+p_{bb}-{\left(p_{BB}-p_{bb}\right)}^{2}]}^{-1}p_{Bb}p_{bb}\\ 4{\left[p_{BB}+p_{bb}-{\left(p_{BB}-p_{bb}\right)}^{2}\right]}^{-1}p_{BB}p_{bb}\;\;\;\mathrm{for}\;\mathrm{genotypes}\;\{\begin{array}{c} BB\\ Bb\\ bb \end{array}\\ {-2\left[p_{BB}+p_{bb}-{\left(p_{BB}-p_{bb}\right)}^{2}\right]}^{-1}p_{BB}p_{Bb} \end{array}$$

according to Vitezica *et al.* (2017). This is different from the D matrix proposed by Su *et al.* (2012) which does not correctly model dominance deviations (and captures part of the GCA), and it is also different from the D matrix in Vitezica *et al.* (2013) (which assumes HWE). The additive-by-additive epistatic relationship matrix can be written as

$$G_{AA^{\left(H\right)}}=\frac{G_{A^{\left(H\right)}}⊙G_{A^{\left(H\right)}}}{tr(G_{A^{\left(H\right)}}⊙G_{A^{\left(H\right)}})/n}$$

### Sub models and model comparison

Variance components were estimated for nested models (GCA-model and G-model) that added, in succession, additive effects ($g_{A}$), dominance effects ($g_{A}+g_{D}$), additive-by-additive genetic effects ($g_{A}+g_{D}+g_{AA}$) in addition to “residual genetic” *r* effects of lines in the GCA-model. The additive-by-additive epistatic effects can be interactions of loci within line from population 1 $g_{{\left(AA\right)}^{\left(1,1\right)}}$, within line from population 2 $g_{{\left(AA\right)}^{\left(2,2\right)}}$ and interactions between loci across lines from populations 1 and 2 $g_{{\left(AA\right)}^{\left(1,2\right)}}$, or within hybrids $g_{{\left(AA\right)}^{H}}$. For details, see Table 2. Also, the variance attributable to GCAs is $\sigma_{GCA^{\left(1\right)}}^{2}=\sigma_{A^{\left(1\right)}}^{2}+\sigma_{{AA}^{\left(1,1\right)}}^{2}+\sigma_{r^{\left(1\right)}}^{2}$, $\sigma_{GCA^{\left(2\right)}}^{2}=\sigma_{A^{\left(2\right)}}^{2}+\sigma_{{AA}^{\left(2,2\right)}}^{2}+\sigma_{r^{\left(2\right)}}^{2}$ and for SCAs is $\sigma_{\mathrm{SCA}}^{2}=\sigma_{D}^{2}+\sigma_{{AA}^{\left(1,2\right)}}^{2}$. Also, another set of models was identical but “residual genetic” *r* effects of lines were not fit in any of the models.

Goodness-of-fit of models was compared based on the deviance information criterion (DIC), which balances model fit and model complexity to avoid overfitting (Spiegelhalter *et al.* 2002). The lower the DIC value, the better fit of the model to the data.

Predicted ability of phenotypes of “untested hybrids” for the different models was tested performing a T2-T1-T0 cross-validation scheme as in Technow et al. (2014). The prediction accuracy of T2, T1, and T0 hybrids (two, one and zero parents, respectively) was obtained for 300 hybrids (N_D_ = 90 and N_F_ = 53 of Dent and Flint parental lines) in the training set. Predictive performance of hybrids was computed separately for each group of hybrids by dividing the correlation of predicted and observed values by $\sqrt{H^{2}}$. $H^{2}$ is the genomic broad-sense heritability. The $H^{2}$ estimated with the full GCA-model was used. The cross-validation process was repeated 100 times.

Estimation of variance components and cross-validation were performed in a Bayesian approach using the BGLR R-package (Pérez and de los Campos 2014). To speed up computation, the eigenvalue decomposition of the variance-covariance matrices was done according to Acosta-Pech *et al.* (2017) and modeled as Bayesian Ridge Regression (BRR). For each model, inferences were based on 30,000 samples collected from 60,000 iterations after discarding 30,000 for burn-in and thinning of 10. Convergence of variance parameters was inspected by trace plots and convergence diagnostic was assessed using the BOA R-package (Smith 2007).

### Data availability

The data set from the breeding program of the University of Hohenheim is available with the publication of Technow *et al.* 2014 (https://doi.org/10.1534/genetics.114.165860). Estimation of variance components and cross-validation were performed in a Bayesian approach using the BGLR R-package (Pérez and de los Campos 2014). The software can be downloaded from https://cran.r-project.org/web/packages/BGLR/index.html. A program to build the genomic matrices is available at http://genoweb.toulouse.inra.fr/~zvitezic/maize.

## Results

### Variance components estimates and heritabilities

Variance components and broad-sense heritabilities ($H^{2}$) for GCA- and G-models are shown in Tables 3 and 4, and for the model without “residual genetic” r effects, in Table A2.

**Table 2:** Definition of genomic models for maize single-cross hybrids

				Variances		
Models	Effects	Model Code	Additive	Dominance	Epistasis	Residual genetic
GCA	$g_{A^{\left(1\right)}}+g_{A^{\left(2\right)}}+r$	GCA:A	$\sigma_{A^{\left(1\right)}}^{2},\sigma_{A^{\left(2\right)}}^{2}$			$\sigma_{r^{\left(1\right)}}^{2},\sigma_{r^{\left(2\right)}}^{2}$
	$g_{A^{\left(1\right)}}+g_{A^{\left(2\right)}}+g_{D}+r$	GCA:AD	$\sigma_{A^{\left(1\right)}}^{2},\sigma_{A^{\left(2\right)}}^{2}$	$\sigma_{D}^{2}$		$\sigma_{r^{\left(1\right)}}^{2},\sigma_{r^{\left(2\right)}}^{2}$
	$g_{A^{\left(1\right)}}+g_{A^{\left(2\right)}}+g_{AA^{\left(1,2\right)}}+r$	$\mathrm{GCA}:A{\left(AA\right)}^{\left(1,2\right)}$	$\sigma_{A^{\left(1\right)}}^{2},\sigma_{A^{\left(2\right)}}^{2}$		$\sigma_{AA^{\left(1,2\right)}}^{2}$	$\sigma_{r^{\left(1\right)}}^{2},\sigma_{r^{\left(2\right)}}^{2}$
	$g_{A^{\left(1\right)}}+g_{A^{\left(2\right)}}+g_{D}+g_{AA^{\left(1,2\right)}}+r$	$\mathrm{GCA}:AD{\left(AA\right)}^{\left(1,2\right)}$	$\sigma_{A^{\left(1\right)}}^{2},\sigma_{A^{\left(2\right)}}^{2}$	$\sigma_{D}^{2}$	$\sigma_{AA^{\left(1,2\right)}}^{2}$	$\sigma_{r^{\left(1\right)}}^{2},\sigma_{r^{\left(2\right)}}^{2}$
	$g_{A^{\left(1\right)}}+g_{A^{\left(2\right)}}+g_{D}+g_{AA^{\left(1,1\right)}}+$ $+g_{AA^{\left(2,2\right)}}+g_{AA^{\left(1,2\right)}}+r$	$\mathrm{GCA}:AD{\left(AA\right)}^{\left(1,1\right)}{\left(AA\right)}^{\left(2,2\right)}{\left(AA\right)}^{\left(1,2\right)}$	$\sigma_{A^{\left(1\right)}}^{2},\sigma_{A^{\left(2\right)}}^{2}$	$\sigma_{D}^{2}$	$\sigma_{AA^{\left(1,1\right)}}^{2},\sigma_{AA^{\left(1,2\right)}}^{2}$ $,\sigma_{AA^{\left(2,2\right)}}^{2}$	$\sigma_{r^{\left(1\right)}}^{2},\sigma_{r^{\left(2\right)}}^{2}$
G	$g_{A^{\left(H\right)}}$	G:A	$\sigma_{A^{\left(H\right)}}^{2}$			
	$g_{A^{\left(H\right)}}+g_{D^{\left(H\right)}}$	$G:AD^{\left(H\right)}$	$\sigma_{A^{\left(H\right)}}^{2}$	$\sigma_{D^{\left(H\right)}}^{2}$		
	$g_{A^{\left(H\right)}}+g_{{AA}^{\left(H\right)}}$	$G:A{\left(AA\right)}^{\left(H\right)}$	$\sigma_{A^{\left(H\right)}}^{2}$		$\sigma_{AA^{\left(H\right)}}^{2}$	
	$g_{A^{\left(H\right)}}+g_{D^{\left(H\right)}}+g_{{AA}^{\left(H\right)}}$	$G:AD^{\left(H\right)}{\left(AA\right)}^{\left(H\right)}$	$\sigma_{A^{\left(H\right)}}^{2}$	$\sigma_{D^{\left(H\right)}}^{2}$	$\sigma_{AA^{\left(H\right)}}^{2}$	

GCA-model (effects (g) and variances ($\sigma^{2}$) defined within heterotic group): additive ($A^{\left(1\right)}$ and $A^{\left(2\right)}$), dominance (D), “residual genetic” (r) and additive-by-additive epistasis (AA) within heterotic groups ((1,1) and (2,2)) and between heterotic groups (1,2).

All the models detailed above were also run without the “residual genetic” r effect term.

**Table 3:** Estimated posterior means and standard deviation (in parenthesis) of genetic variance components obtained with two genomic models for maize grain yield

Model Code	Additive		Dominance	Epistasis			Residual genetic		Residual
	$\sigma_{A^{\left(1\right)}}^{2},\sigma_{A^{\left(2\right)}}^{2}$ or $\sigma_{A^{\left(H\right)}}^{2}$		$\sigma_{D}^{2}$ or $\sigma_{D^{\left(H\right)}}^{2}$	$\sigma_{AA^{\left(1,1\right)}}^{2}$	$\sigma_{AA^{\left(2,2\right)}}^{2}$	$\sigma_{AA^{\left(1,2\right)}}^{2}$ or $\sigma_{AA^{\left(H\right)}}^{2}$	$\sigma_{r^{\left(1\right)}}^{2}$	$\sigma_{r^{\left(2\right)}}^{2}$	$\sigma_{e}^{2}$
GCA:A	23.16 (4.78)	12.92 (3.49)					6 (1.59)	6.47 (1.81)	17.63 (0.77)
GCA:AD	22.97 (4.67)	13.07 (3.5)	3.59 (0.72)				5.2 (1.47)	5.9 (1.71)	15.01 (0.79)
$\mathrm{GCA}:A{\left(AA\right)}^{\left(1,2\right)}$	22.87 (4.75)	13.05 (3.55)				4.75 (0.93)	5.13 (1.43)	5.89 (1.72)	13.8 (0.84)
$\mathrm{GCA}:AD{\left(AA\right)}^{\left(1,2\right)}$	22.82 (4.84)	13.02 (3.46)	2.48 (0.54)			3.6 (0.86)	4.84 (1.45)	5.51 (1.66)	13.46 (0.82)
$\mathrm{GCA}:AD{\left(AA\right)}^{\left(1,1\right)}{\left(AA\right)}^{\left(2,2\right)}{\left(AA\right)}^{\left(1,2\right)}$	19.06 (4.78)	10.61 (3.48)	2.3 (0.56)	5.41 (2.01)	5.57 (2.15)	3.24 (0.79)	3.8 (1.23)	4.56 (1.56)	13.67 (0.81)
G:A	51.77 (6.75)								18.02 (0.79)
$G:AD^{\left(H\right)}$	47.81 (6.35)		6.18 (1.06)						14.97 (0.78)
$G:A{\left(AA\right)}^{\left(H\right)}$	42.22 (6.30)					10.2 (1.76)			13.89 (0.82)
$G:AD^{\left(H\right)}{\left(AA\right)}^{\left(H\right)}$	42.26 (6.08)		4.14 (0.81)			7.19 (1.55)			13.59 (0.80)

Estimates of additive ($\sigma_{A^{\left(1\right)}}^{2},\sigma_{A^{\left(2\right)}}^{2}$ or $\sigma_{A^{\left(H\right)}}^{2}$), dominance ($\sigma_{D}^{2}$ or $\sigma_{D^{\left(H\right)}}^{2}$), additive-by-additive ($\sigma_{AA^{\left(1,1\right)}}^{2}$, $\sigma_{AA^{\left(2,2\right)}}^{2}$, $\sigma_{AA^{\left(1,2\right)}}^{2}$ or $\sigma_{AA^{\left(H\right)}}^{2}$), residual genetic effects ($\sigma_{r^{\left(1,1\right)}}^{2}$, $\sigma_{r^{\left(2,2\right)}}^{2}$) and residual ($\sigma_{e}^{2}$) variances for GCA- and G-models and successively added additive effects (A), dominance effects (AD), additive-by-additive effects (AD(AA)). Superscripts 1 and 2 in parenthesis refers to dent and flint heterotic groups, respectively. The additive-by-additive epistatic effects can be interactions between loci within group (${\left(AA\right)}^{\left(11\right)}$ and ${\left(AA\right)}^{\left(22\right)}$), across groups ${\left(AA\right)}^{\left(12\right)}$ or within hybrids ${\left(AA\right)}^{\left(H\right)}$.

We consider first Table 3 and the GCA-model. The total variance of GCA *e.g.* for population 1 is $\sigma_{GCA^{\left(1\right)}}^{2}=\sigma_{A^{\left(1\right)}}^{2}+\sigma_{{AA}^{\left(1,1\right)}}^{2}+\sigma_{r^{\left(1\right)}}^{2}$, and this changes very little across models: total GCA variance for population 1 oscillates between 27.66 and 29.16, and for population 2 between 18.53 and 20.74. Within GCA, each individual component varies across the different models but changes are not large; the major change is the diminution from 23.16 to 19.06 in the estimate of $\sigma_{A^{\left(1\right)}}^{2}$ when $\sigma_{{AA}^{\left(1,1\right)}}^{2}$ is fit, and similarly ($\sigma_{A^{\left(2\right)}}^{2}$ changes from 12.92 to 10.61) for population 2. This is due to reassignment of total GCA variance across its component parts; the r effect does not fully account for the absence of the $\sigma_{{AA}^{\left(1,1\right)}}^{2}$ variance component.

As for the SCA, its two components ($\sigma_{D}^{2}$ and $\sigma_{{AA}^{\left(1,2\right)}}^{2}$) also show some changes and there is some reassignment from one to the other. Still, for GCA and SCA components changes are not great and enter well within the confidence intervals of the estimates.

On the contrary, when “residual genetic” r effects were not fit (Table A2), changes were much larger. In particular, the inclusion of within-group epistatic variances ($\sigma_{{AA}^{\left(1,1\right)}}^{2}$ and $\sigma_{{AA}^{\left(2,2\right)}}^{2}$) reduced additive variances from 31.08 to 22.30 for $\sigma_{A^{\left(1\right)}}^{2}$, and from 22.11 to 14.42 for $\sigma_{A^{\left(2\right)}}^{2}$ in the full model.

Thus, we conclude that the GCA-model is reasonably (empirically) orthogonal when “residual genetic effects” for GCA of lines are fit, regardless of the level of complexity for the genetic modelling (additive, additive by additive, etc.). Using “residual genetic effects” allows to accommodate non-additive effects of lines not explicitly modelled.

Some of these changes can be attributed to similarity of relationship matrices. The correlation between $G_{A^{\left(1\right)}}$ and $G_{{\mathrm{AA}}^{\left(1,1\right)}}$ (and regression coefficient of $G_{A^{\left(1\right)}}∼G_{AA^{\left(1,1\right)}}$) was 0.39 (0.92); and the correlation between $G_{A^{\left(2\right)}}$ and $G_{{AA}^{\left(2,2\right)}}$ (and regression coefficient of $G_{A^{\left(2\right)}}∼G_{AA^{\left(2,2\right)}}$) was 0.55 (1.63). These results show a high similarity between these relationship matrices and explain that the additive effects tend to capture additive by additive effects if the latter are not explicitly fit (as described above and shown in Table A2), something that is ameliorated fitting “residual genetic” r effects (Table 3).

Similarly, D and $G_{{AA}^{\left(1,2\right)}}$ matrices were highly correlated (0.88), suggesting that it is difficult to accurately separate dominance and across-groups additive by additive epistasis. To avoid redundancy between the D and $G_{{AA}^{\left(1,2\right)}}$ matrices, we corrected the $G_{{AA}^{\left(1,2\right)}}$ matrix by subtracting the contribution of dominance as in Alves *et al.* (2019). However, the resulting $G_{{AA}^{\left(1,2\right)}}$had a similar correlation to D. Also the regression coefficient of $D∼G_{AA^{\left(1,2\right)}}$ was 1.02, indicating that elements of $G_{AA^{\left(1,2\right)}}$ are unbiased (but shrunken) estimators of the elements of D.

For the G-model, Table 3 shows that the additive variance estimate in the G:A model (51.77) was higher than the addition of additive variance estimates for Dent and Flint groups (36.08) in the GCA:A model. Similarly, estimates of dominance ($\sigma_{D^{\left(H\right)}}^{2}$) and epistasis within hybrids ($\sigma_{AA^{\left(H\right)}}^{2}$) variances were higher than in GCA-models. Both results are in agreement with Stuber and Cockerham (1966). The inclusion of the additive-by-additive epistasis effects in the $G:AD{\left(AA\right)}^{\left(H\right)}$ model reduced the estimate of additive variance ($\sigma_{A^{\left(H\right)}}^{2}$) from 51.77 to 42.26. Furthermore, similar to GCA-models, the sum of estimates of $\sigma_{D^{\left(H\right)}}^{2}$ and $\sigma_{AA^{\left(1,2\right)}}^{2}$ (11.33) obtained with the $G:AD^{\left(H\right)}{\left(AA\right)}^{\left(H\right)}$ model was lower than the sum (16.38) of the estimates of $\sigma_{D^{\left(H\right)}}^{2}$ (in the $G:AD^{\left(H\right)}$ model) and of $\sigma_{AA^{\left(H\right)}}^{2}$ (in the $G:A{\left(AA\right)}^{\left(H\right)}$). In the G-model, it is impossible to include “residual genetic” r effects because the model is fit at the hybrid and not at the line (GCA) level, and in this data set there is a single record per hybrid. Thus we conclude that the G-model, although constructed with an orthogonal formalism, is not empirically orthogonal with this data set. These results can also be explained because there was a correlation of 0.55 between $G_{A^{\left(H\right)}}$ and $G_{AA^{\left(H\right)}}$ and of 0.67 between $D_{H}$ and $G_{AA^{\left(H\right)}}$.

Estimates of genomic broad-sense heritability $H^{2}$ for grain yield ranged from 0.73 to 0.80, and from 0.74 to 0.80 in the full GCA- and G-models, respectively (Table 4). Estimates of residual variances were similar between GCA-models and G-models (Table 3). The estimates of residual variances decreased as the non-additive genetic effects were added in both GCA- and G-models.

### Goodness of fit

Table 4 shows the Deviance Information Criteria (DIC) values for each model. Inclusion of non-additive effects in GCA- and G- models improved the goodness of fit of both models. DIC values between GCA- and G-models were very similar. Among all models, the best model (with lower DIC value) was the $\mathrm{GCA}:A{\left(AA\right)}^{\left(1,2\right)}$. Among the G-models, the best one was that accounted only for additive and additive-by-additive epistatic effects. Models including both dominance and epistatic effects had slightly worse DIC values than the best one. This can be explained because increasing the number of parameters may lead to overfitting and thus, it penalizes DIC values.

Based on the inspection of the trace plot and convergence diagnostic with BOA R-package (Smith 2007), all estimates of the variance parameters in all models converged to the posterior distribution.

### Cross-validation

The results for cross-validation are shown in Table 5. In general, prediction accuracy was considerably high for maize grain yield (>0.80 in all cases) and similar values were obtained with the G- and GCA- models in this data set. The inclusion of non-additive genetic effects did not improve the prediction accuracy of hybrid values in the testing sets compared to models including only additive effects. The only factor that counted in the predictive ability was the fact of having both, one, or no parents in the training data set, with respective prediction accuracies 0.80, 0.88 and 0.92.

**Table 4:** Estimated posterior means and standard deviation (in parenthesis) of broad-sense heritability and Deviance Information Criteria (DIC) values obtained with two genomic models for maize grain yield

Model Code	$H^{2}$	DIC
GCA:A	0.73 (0.05)	7321.5
GCA:AD	0.77 (0.04)	7254.27
$\mathrm{GCA}:A{\left(AA\right)}^{\left(1,2\right)}$	0.79 (0.04)	7201.91
$\mathrm{GCA}:AD{\left(AA\right)}^{\left(1,2\right)}$	0.79 (0.05)	7203.69
$\mathrm{GCA}:AD{\left(AA\right)}^{\left(1,1\right)}{\left(AA\right)}^{\left(2,2\right)}{\left(AA\right)}^{\left(1,2\right)}$	0.80 (0.04)	7209.92
G:A	0.74 (0.03)	7335.75
$G:AD^{\left(H\right)}$	0.78 (0.02)	7260.88
$G:A{\left(AA\right)}^{\left(H\right)}$	0.78 (0.02)	7206.89
$G:AD^{\left(H\right)}{\left(AA\right)}^{\left(H\right)}$	0.80 (0.02)	7212.46

GCA- and G-models are models that successively added additive effects (A), dominance effects (AD), and additive-by-additive genetic effects (AD(AA)). The additive-by-additive epistatic effects can be interactions between loci within group (${\left(AA\right)}^{\left(11\right)}$ and ${\left(AA\right)}^{\left(22\right)}$), across groups ${\left(AA\right)}^{\left(12\right)}$ or within hybrids ${\left(AA\right)}^{\left(H\right)}$. Superscripts 1 and 2 in parenthesis refers to dent and flint heterotic groups, respectively. $H^{2}$ is the genomic broad-sense heritability.

**Table 5:** Predictive accuracy of T2, T1 and T0 hybrids obtained with two genomic models for maize grain yield

Model Code	T2	T1	T0
GCA:A	0.92 (0.03)	0.88 (0.02)	0.80 (0.08)
GCA:AD	0.92 (0.03)	0.88 (0.02)	0.80 (0.08)
$\mathrm{GCA}:A{\left(AA\right)}^{\left(1,2\right)}$	0.92 (0.03)	0.88 (0.02)	0.80 (0.08)
$\mathrm{GCA}:AD{\left(AA\right)}^{\left(1,2\right)}$	0.92 (0.03)	0.88 (0.02)	0.80 (0.08)
$\mathrm{GCA}:AD{\left(AA\right)}^{\left(1,1\right)}{\left(AA\right)}^{\left(2,2\right)}{\left(AA\right)}^{\left(1,2\right)}$	0.92 (0.03)	0.88 (0.02)	0.80 (0.08)

G:A	0.92 (0.03)	0.88 (0.03)	0.81 (0.08)
$G:AD^{\left(H\right)}$	0.92 (0.03)	0.88 (0.03)	0.81 (0.08)
$G:A{\left(AA\right)}^{\left(H\right)}$	0.92 (0.03)	0.89 (0.03)	0.81 (0.08)
$G:AD^{\left(H\right)}{\left(AA\right)}^{\left(H\right)}$	0.92 (0.03)	0.88 (0.03)	0.81 (0.08)

GCA- and G-models are models that successively added additive effects (A), dominance effects (AD), and additive-by-additive genetic effects (AD(AA)). The additive-by-additive epistatic effects can be interactions between loci within group (${\left(AA\right)}^{\left(11\right)}$ and ${\left(AA\right)}^{\left(22\right)}$), across groups ${\left(AA\right)}^{\left(12\right)}$ or within hybrids ${\left(AA\right)}^{\left(H\right)}$. Superscripts 1 and 2 in parenthesis refers to dent and flint heterotic groups, respectively. The values refer to the mean (standard deviation) over 100 cross-validation runs with the different models. For T2, T1 and T0 group hybrids, two, one and zero parents were tested in the training set.

## Discussion

In this study, the theory in the analysis of hybrid crosses of inbred lines from two populations using relationship matrices was revisited in a genomic context. Models for genomic prediction in hybrid crops using the notions of effects defined “according to origin” (GCAs and SCAs) were rederived and expressions for additive, dominant and epistatic relationships for hybrids were presented. These models were applied to a public data set to exemplify the theory and its consequences in real life.

### Insights into relationships for dominance and across-population pairwise epistasis

A surprising fact (to us) is that, in the classical pedigree-based methods, it is not possible to disentangle dominance deviations from across-population epistasis, whereas using markers it is possible. This seems not to have been recognized by previous researchers, leading to the wrong conclusion that in a genomic setting the relationship of dominance deviations is a product of corresponding additive relationships of parental lines. In this section we try to explain why such a difference.

Stuber and Cockerham (1966) used the notion of identity by descent (IBD) coefficients to model relationships, where the starting block is the use of coancestries Φ - the probability that two alleles drawn at random from each of two pure lines are identical by descent. Although in our work we use genomic relationships (that are not probabilities), the concept of IBD is useful in the following.

**Figure 1:**
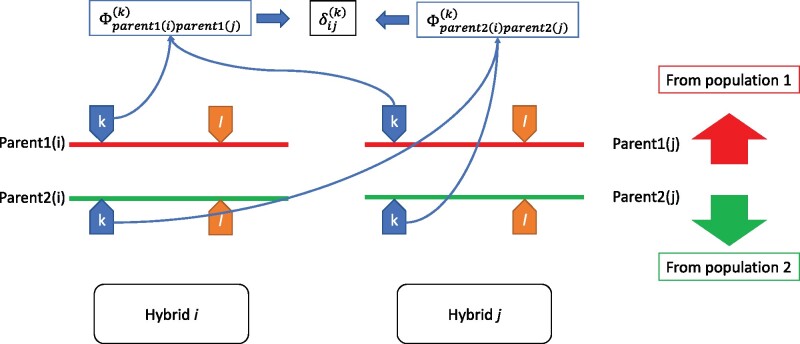
Dominance relationship across two hybrids for locus k.

For two hybrids, the IBD dominance relationship coefficient at locus *k* (say $\delta^{\left(k\right)}$) is the probability that two complete genotypes at locus *k* in hybrids (*i* and *j*) are identical, and because the lines are fully inbred, this is the joint probability that both “parents1” (ancestors from population 1) are IBD at locus *k* (with probability $\Phi_{\mathrm{parent}1\left(i\right)p\mathrm{arent}1\left(j\right)}^{\left(k\right)}$) and both “parents2” (ancestors from population 2) are IBD at locus *k* (with probability $\Phi_{\mathrm{parent}2\left(i\right)p\mathrm{arent}2\left(j\right)}^{\left(k\right)}$) (see Figure 1). This results in $\delta_{ij}^{\left(k\right)}=\Phi_{\mathrm{parent}1\left(i\right)p\mathrm{arent}1\left(j\right)}^{\left(k\right)}\Phi_{\mathrm{parent}2\left(i\right)p\mathrm{arent}2\left(j\right)}^{\left(k\right)}$. Across all *m* loci, $\delta_{ij}=\frac{1}{m}\sum_{k=1,m}\delta_{ij}^{\left(k\right)}=\frac{1}{m}\sum_{k=1,m}\left(\Phi_{\mathrm{parent}1\left(i\right)p\mathrm{arent}1\left(j\right)}^{\left(k\right)}\Phi_{\mathrm{parent}2\left(i\right)p\mathrm{arent}2\left(j\right)}^{\left(k\right)}\right)$. However, in practice, pedigree-based coancestries at specific loci are not observable and they are replaced by infinitesimal coancestries:

$$\delta_{ij}=\frac{1}{m}\sum_{k=1,m}\left(\Phi_{\mathrm{parent}1\left(i\right)p\mathrm{arent}1\left(j\right)}^{\left(k\right)}\Phi_{\mathrm{parent}2\left(i\right)p\mathrm{arent}2\left(j\right)}^{\left(k\right)}\right)\approx \frac{1}{m}\sum_{k=1,m}\left(\Phi_{\mathrm{parent}1\left(i\right)p\mathrm{arent}1\left(j\right)}\;\Phi_{\mathrm{parent}2\left(i\right)p\mathrm{arent}2\left(j\right)}\;\right)=\Phi_{par\mathrm{ent}1\left(i\right)p\mathrm{arent}1\left(j\right)}\;\Phi_{\mathrm{parent}2\left(i\right)p\mathrm{arent}2\left(j\right)}$$

resulting in $\delta_{ij}\approx \Phi_{\mathrm{parent}1\left(i\right)p\mathrm{arent}1\left(j\right)}\;\Phi_{\mathrm{parent}2\left(i\right)p\mathrm{arent}2\left(j\right)}\;$which is the expression presented by Stuber and Cockerham (1966). The approximation results from the fact that the genome is finite. For instance, if there were m = 3 loci, there would be 3 local IBD, one at each locus, whose average will not in general be the same as the pedigree-based IBD which does assume infinite loci (Hill and Weir 2011). In an infinitesimal model, the approximation is exact.

**Figure 2:**
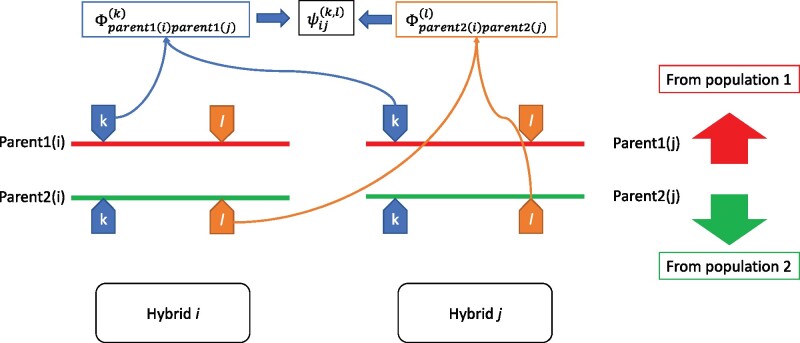
Additive by additive epistatic across-population relationship across two hybrids for locus k and l.

Now we address the across-population epistatic additive by additive relationships. Consider two loci *k* and *l*. In an IBD framework, across-population epistatic additive by additive relationship for hybrids *i* and *j* at two loci *k* and *l* (say $\psi_{ij}^{\left(k,l\right)}$) is the joint probability that both “parents1” (ancestors from population 1) have the same genotype at locus *k* and that both “parents2” (ancestors from population 2) have the same genotype at locus *l* (see Figure 2). Thus, $\psi_{ij}^{\left(k,l\right)}=\Phi_{\mathrm{parent}1\left(i\right)p\mathrm{arent}1\left(j\right)}^{\left(k\right)}\Phi_{\mathrm{parent}2\left(i\right)p\mathrm{arent}2\left(j\right)}^{\left(l\right)}$. Whole-genome epistatic relationship would therefore be

$$\psi_{ij}=\frac{1}{m\left(m-1\right)}\sum_{k,l,k\neq l}\Phi_{\mathrm{parent}1\left(i\right)p\mathrm{arent}1\left(j\right)}^{\left(k\right)}\Phi_{\mathrm{parent}2\left(i\right)p\mathrm{arent}2\left(j\right)}^{\left(l\right)}$$

However, based on pedigree, the different $\Phi_{\mathrm{parent}1\left(i\right)p\mathrm{arent}1\left(j\right)}^{\left(l\right)}$ and $\Phi_{\mathrm{parent}2\left(i\right)p\mathrm{arent}2\left(j\right)}^{\left(k\right)}$ are not observable and they are replaced by infinitesimal coancestries resulting in the approximation $\psi_{ij}\approx \Phi_{\mathrm{parent}1\left(i\right)p\mathrm{arent}1\left(j\right)}\;\Phi_{\mathrm{parent}2\left(i\right)p\mathrm{arent}2\left(j\right)}$. Again, in an infinitesimal model the approximation is exact.

It is worth noting that $\psi_{ij}=\frac{1}{m\left(m-1\right)}\sum_{k,l,k\neq l}\Phi_{\mathrm{parent}1\left(i\right)p\mathrm{arent}1\left(j\right)}^{\left(l\right)}\Phi_{\mathrm{parent}2\left(i\right)p\mathrm{arent}2\left(j\right)}^{\left(k\right)}$ involves relationships *across pairs* of loci whereas $\delta_{ij}=\frac{1}{m}\sum_{k=1,m}\left(\Phi_{\mathrm{parent}1\left(i\right)p\mathrm{arent}1\left(j\right)}^{\left(k\right)}\Phi_{\mathrm{parent}2\left(i\right)p\mathrm{arent}2\left(j\right)}^{\left(k\right)}\right)$ involves relationships *within single* loci. On average, pairs of loci are transmitted in a manner similar to transmission of single locus (for instance two neighboring markers are often transmitted together), which explains why $\psi_{ij}$ is an estimator (albeit not necesssarily a good one) of $\delta_{ij}$, and it explains why the elements of $G_{AA^{\left(1,2\right)}}$ are unbiased (but not necessarily accurate) estimators of the elements of D, as shown by the results.

Thus, we have shown that the Stuber and Cockerham (1966) relationships assuming pedigrees are only exact under infinitesimal models. In previous sections we have shown that observing the genome (*i.e.* with markers), different relationships can be formed for each, additive substitution and dominant and epistatic deviations. Thus, contrary to pedigree-based formulations, a marker-based formulation allows disentangling of the different variance components.

Thus, in pedigree-based models the dominance and across-population epistatic relationships are conceptually different, but the lack of other information forces to use the same estimator for both. This is not the case in marker-based models, where we can actually observe different relationships within locus or across loci from two populations.

### Partition of genetic variance components and heritability

The partition of the genetic variance in terms of statistical additive effects, and dominance and epistatic deviations effects, was possible using the relationship matrices developed here. In our model, estimates of additive genetic variance based on allele substitution effects are useful for selection or in the prediction of potential selection response in pool improvement. Vitezica *et al.* (2013) compared a classical model (in terms of statistical values for breeding purposes) with a genotypic model (biological values at the gene level) proposed by Su *et al.* (2012). When the genotypic model is used, additive and dominant genotypic variances are obtained. Both models are able to explain the data but their results and interpretation is different (Vitezica *et al.* 2013; Varona *et al.* 2018). The genotypic model has been used for hybrid genomic prediction (Fristche-Neto *et al.* 2018; Werner *et al.* 2018; Alves *et al.* 2019; Ramstein *et al.* 2020), but estimates of genotypic additive variance should not be interpreted for breeding purposes. The GCA- (proposed here) and G-models are equivalent models to explain the data only if all relevant gene actions (*i.e.* high order interactions) are included (Stuber and Cockerham 1966), but it is impossible to ascertain if all relevant interactions are included. In our results, both definitional systems perform similarly for prediction. However, as the G-model assumes gene effects uniquely within hybrids and does not provide additive values within pool, it can not be directly used for the selection of inbred lines within pools for recurrent pool improvement. Thus the GCA-model is more useful.

Orthogonal partitioning of the effects has been described extensively (*e.g.*Cockerham 1954; Kempthorne,1954; Lynch and Walsh 1998) for classical HWE populations but also for hybrid crosses (*e.g.*Griffing 1962; Stuber and Cockerham 1966; Bernardo 1996). Statistically, orthogonality means that inclusion of new terms in the model does not change the definition (in practical terms: the estimates) of already included effects in an ideal, infinitely large population. For instance, by construction, in an orthogonal model there is no covariance across statistical additive and dominance effects. This implies that the covariance across hybrids can be split in covariance due to additive effects, covariance due to dominance deviations, and so on (Lynch and Walsh 1998). Another advantage of using orthogonality in Genetics and breeding is the interpretability. It is the only way to carry out the estimation of GCA (additive “statistical” effects + within-group epistatic “statistical” interactions) in an unambiguous manner, *i.e.* such that their definitions do not depend on other genetic terms that are fitted in the model.

In practice, additive, epistatic and other variances can not be accurately disentangled with a small data set and many (unknown) QTL loci. However, even with a thousand records and thirty thousand markers (as in this work), it still makes sense to orthogonally define the genetic effects in the model. Not using orthogonal partitions might lead to ambiguous definitions of effects and to potential mistakes. For instance, if the additive variance is inflated, a possible consequence is that the genetic progress can be overestimated. If the dominance variance is inflated, the role of assortative mating of pairs of lines to produce a hybrid could be exaggerated. In our work we used orthogonal definitions of effects and the corresponding relationship matrices, as well as “residual genetic” r effects to account for unmodelled higher-order effects. In this manner we obtained, in the GCA-model, empirically orthogonal estimates of additive, dominance and epistatic variances for maize grain yield.

In the GCA-model, after fitting the “residual genetic” r effect, additive variances were similar across different models (∼22 and ∼12 for group 1 and 2, respectively: see Table 3) showing empirical orthogonality (Hill and Mäki‐Tanila 2015; Vitezica *et al.* 2017). For planning the breeding scheme (to estimate genetic gain and selection of within pools crosses), it is important to obtain good estimates of the genetic variance, and therefore we recommend fitting “residual genetic” r effects, in order to avoid overestimation of the genetic additive variance. The latter option is only possible if each line contributes to several phenotyped hybrids.

In the G-model, when within-group epistatic effects were not fitted, additive variance was overestimated. Similar results were observed by Bernardo (1995). He attributed this to multicollinearity between the additive and within-group epistatic relationships, as we observe. Working with repeated measures per individual, Vitezica *et al.* (2018) fitted a G-model with “residual genetic” r effects and they obtained empirically unbiased estimates of additive variance. However, in the present work it was not possible to fit “residual genetic” hybrid effects in the G-model because in our dataset each hybrid has a single record (adjusted entry means).

Genomic relationship matrices for within and across groups epistasis (in the full GCA-model) allows to partition the genetic variance in terms of GCA and SCA effects, as was originally defined by Stuber and Cockerham (1966) in an infinitesimal context. With our model, it is possible to split the GCA effect into the additive gametic effect and the additive-by-additive epistasis interaction within the line; and split the SCA effect into dominance deviation effect and additive-by-additive epistasis across groups. This has practical implications in hybrid breeding programs that will be discussed later.

Compared to the estimates of genetic variance component from Technow *et al.* (2014), we obtained similar estimates with the $\mathrm{GCA}:\;A{\left(\mathrm{AA}\right)}^{\left(1,2\right)}$ model (see Table 3). This makes sense because, as indicated in the theory, the estimate of SCA variation from Technow *et al.* (2014), is in fact the estimate of epistasis variation across populations $\sigma_{AA^{\left(1,2\right)}}^{2}$. Their entry-mean heritability was 0.87, whereas our genomic estimate of broad-sense heritability was slightly lower (0.81). Differences between our and their estimates are mainly because we used entry means (publicly available) instead of the whole data set, which can be seen through the estimated residual variance which was much lower (*e.g.* ∼17) than their estimated values (179).

### Goodness of fit

Models with lower DIC values better fit the data, and a difference less than 7 units is often considered as irrelevant (Plummer *et al.* 2006). In general, the inclusion of non-additive genetic effects improved the goodness of fit to the data in both GCA- and G- models in this set of hybrids. This result agrees with previous studies in maize hybrids ( Ferrão *et al.* 2020; Alves *et al.* 2019; Hunt *et al.* 2020). DIC values obtained with the GCA-model were similar to those obtained in G-models, indicating that they are equivalent models in terms of fitting the data. The best model, with a best balance between goodness of fit and model complexity, was the $\mathrm{GCA}:A{\left(AA\right)}^{\left(1,2\right)}$, which corresponds to a frequently used model in genomic prediction of hybrids (Technow *et al.* 2014). That means this model is efficient to fit the data. However, fit to the data is not the only aspect that should be considered—interpretation of the model in a genetic context is important.

### Cross-validation

Overall, cross-validation analyses yielded a high prediction accuracy of hybrid performance (>0.80). This is because a high heritability generally results in high prediction accuracy, as was showed theoretically and empirically (Daetwyler *et al.* 2010; Combs and Bernardo 2013). Inclusion of non-additive genetic effects did not show improvement in prediction accuracy. This result agrees with other studies using real data where virtually no benefit was observed by including SCA effects in genomic prediction models of inter-heterotic-group hybrids (Bernardo 1994; Schrag *et al.* 2006, 2018; Maenhout *et al.* 2010; Kadam *et al.* 2016). This is because in inter-heterotic-group hybrids the proportion of SCA variance is often low and GCA high (Reif *et al.* 2007).

We used the splitting of cross-validation considering T2, T1 and T0 (groups of hybrids with two, one and zero parents known in the training set) as in Technow *et al.* (2014). Our predictive abilities were comparable to those reported by Technow *et al.* (2014). For instance, the correlation obtained with the $\mathrm{GCA}:AD{\left(AA\right)}^{\left(1,2\right)}$ results in values of 0.92, 0.88 and 0.80, which are close to the correlations of 0.91, 0.85 and 0.77 (for 300 hybrids in the training set) for T2, T1 and T0, respectively, reported by Technow *et al.* (2014) for grain yield.

Assuming marker effects defined uniquely at the hybrid level (G-models) gave similar prediction accuracy than assuming gene effects according to origin (GCA-models). This result was also reported by Technow *et al.* (2014) with the same data set, but also by Alves *et al.* (2019) who analyzed a population of hybrids derived from a convergent population. Thus, GCA- and G- models are equivalent in terms of predictive ability of hybrid performance. However, our aim in this work is to introduce a more meaningful model (the GCA-model), and its superiority is not to be considered only in terms of better prediction ability in the hybrids.

### Practical implications in hybrid breeding

The way of partitioning the genetic variance is to a certain extent a matter of convenience. Partitioning in terms of GCA (within group) is more convenient because inbred lines are actually created and selected within group. The magnitude of the GCA variance gives to the breeder an idea of how much overall genetic variation coming from the parents is expected in the hybrids. Further, splitting the GCA variance into additive and epistasis within group is relevant at the moment of planning the genetic progress in maize breeding programs. The genetic improvement in hybrid performance is through the selection of inbred lines. So that, breeders create new segregating (*e.g.* F2) populations by crossing elite lines within groups followed by subsequent generation of inbreeding to develop new inbred lines. Therefore, the particular additive-by-additive (and higher order) epistatic combination existing in a particular elite line is not transmitted as a whole to its F2 (and further selfing) progeny, because meiosis and recombination shuffles alleles of the two parents in the cross, breaking down the original epistatic combinations present in the elite inbred lines and creating new epistatic combinations. Thus, the use of the additive variance, instead of the total GCA variance, is more appropriate for the prediction of genetic progress that is achievable by selecting *within* heterotic pools (Stuber and Cockerham 1966). In addition, variance of epistasis within groups is expected to be converted in new additive genetic variance in the long term by random drift, thus, it affects the long-term selection response indirectly (Hill 2017). Also, for pool improvement, it is better to use estimates of additive effects instead of estimates of GCA, because the first reflect better expected genetic progress.

Splitting the SCA variance into dominance deviations and epistasis across groups could also have practical implications. Estimates of additive and dominance effects might be important for hybrid pool development. For instance, Zhao *et al.* (2015) suggested to use additive and dominance effects from an incomplete factorial in order to develop heterotic pools in wheat. Further, estimates of dominance deviations are relevant in the definition of mate allocation procedures (Varona *et al.* 2018); for instance, they could be used to maximize hybrid performance or maintain diversity for long-term genetic gain in hybrid breeding programs (*e.g.*Allier *et al.* 2019).

In maize, there is evidence of directional dominance (Reif *et al.* 2003; Ramstein *et al.* 2020). Indeed, directional dominance as a biological mechanism should exist, given that hybrids show heterosis. When there is directional dominance (*i.e.* a higher percentage of positive than negative dominance effects, E(d)≠0), overall heterosis could be considered in the genetic evaluation model. If individuals expressing the trait show considerable variation in heterozygosity (*e.g.* in a diallel design with crosses within- and across- groups), a more diverse individual will show more positive heterosis at the trait. De Boer and Hoeschele (1993) showed analytically that not fitting this heterosis (usually as a covariate) leads to spurious overestimation of dominance variation, as shown with real data (Xiang *et al.* 2016, Aliloo *et al.* 2017, Varona *et al.* 2018). Nonetheless, preliminary results in this work showed that heterosis (measured as number of heterozygotic loci) was very similar across hybrids and fitting heterosis in the models led to very similar results (not shown).

## Conclusions

Models developed here, with effects defined according to origin (GCA-), and using genomic relationships properly defined for each statistical component, allow for a proper partition of statistical additive effects, dominance deviations, and epistatic deviations, in hybrids derived from inbred lines from two populations. Contrary to common belief, using SNP genotypes, it is possible to split SCA into dominance deviations and across-groups epistasis, and to split GCA into within-line additive effects and within-line epistatic effects. Our GCA-model is appropriate for genomic prediction and variance component estimation in hybrid crops using genomic data, and its results (estimates of genetic variance components, breeding values and deviations) can be practically interpreted and used for breeding purposes.

## APPENDIX A

### Dominance deviations

The table of genotypic values is

The mean of the genotypic value G of the crossbred population is

$$E\left(G\right)=p_{1}p_{2}\left(a_{1}+a_{2}\right)+p_{1}q_{2}\left(a_{1}+d\right)+q_{1}p_{2}\left(a_{2}+d\right)=p_{1}a_{1}+p_{2}a_{2}+\left(p_{1}q_{2}+q_{1}p_{2}\right)d$$

After centering, the table of centered genotypic values is

The sum of the breeding values of the different gametes at the hybrid is

Subtracting this table from the centered genotypic values gives dominance deviations. If we go genotype by genotype:

$$\delta_{B_{1}B_{2}}=q_{1}a_{1}+q_{2}a_{2}-\left(p_{1}q_{2}+q_{1}p_{2}\right)d-q_{1}\alpha_{1}-q_{2}\alpha_{2}=$$

$$q_{1}a_{1}+q_{2}a_{2}-\left(p_{1}q_{2}+q_{1}p_{2}\right)d-q_{1}\left(a_{1}+\left(q_{2}-p_{2}\right)d\right)-q_{2}\left(a_{2}+\left(q_{1}-p_{1}\right)d\right)=$$

$$-\left(p_{1}q_{2}+q_{1}p_{2}\right)d-q_{1}\left(q_{2}-p_{2}\right)d-q_{2}\left(q_{1}-p_{1}\right)d=$$

$$\left(-p_{1}q_{2}-q_{1}p_{2}-q_{1}q_{2}+q_{1}p_{2}-q_{1}q_{2}+q_{2}p_{1}\right)d=-2q_{1}q_{2}d$$

$$\delta_{B_{1}b_{2}}=q_{1}a_{1}-p_{2}a_{2}+\left(1-p_{1}q_{2}-q_{1}p_{2}\right)d-\left(q_{1}\alpha_{1}-p_{2}\alpha_{2}\right)=$$

$$q_{1}a_{1}-p_{2}a_{2}+\left(1-p_{1}q_{2}-q_{1}p_{2}\right)d-q_{1}\left(a_{1}+\left(q_{2}-p_{2}\right)d\right)+p_{2}\left(a_{2}+\left(q_{1}-p_{1}\right)d\right)=$$

$$\left(1-p_{1}q_{2}-q_{1}p_{2}\right)d-q_{1}\left(q_{2}-p_{2}\right)d+p_{2}\left(q_{1}-p_{1}\right)d=$$

$$\left(1-p_{1}q_{2}-q_{1}p_{2}-q_{1}q_{2}+q_{1}p_{2}+p_{2}q_{1}-p_{1}p_{2}\right)d=$$

$$\left(1-p_{1}\left(q_{2}+p_{2}\right)-q_{1}q_{2}+p_{2}q_{1}\right)d=$$

$$\left(q_{1}-q_{1}q_{2}+p_{2}q_{1}\right)d=q_{1}\left(1-q_{2}+p_{2}\right)d=2q_{1}p_{2}d$$

$$\delta_{b_{1}B_{2}}=q_{2}a_{2}-p_{1}a_{1}+\left(1-p_{1}q_{2}-q_{1}p_{2}\right)d-\left(-p_{1}\alpha_{1}+q_{2}\alpha_{2}\right)=$$

$$q_{2}a_{2}-p_{1}a_{1}+\left(1-p_{1}q_{2}-q_{1}p_{2}\right)d-\left(-p_{1}\left(a_{1}+\left(q_{2}-p_{2}\right)d\right)+q_{2}\left(a_{2}+\left(q_{1}-p_{1}\right)d\right)\right)=$$

$$\left(1-p_{1}q_{2}-q_{1}p_{2}\right)d+p_{1}\left(q_{2}-p_{2}\right)d-q_{2}\left(q_{1}-p_{1}\right)d=$$

$$\left(1-p_{1}q_{2}-q_{1}p_{2}+p_{1}q_{2}-p_{1}p_{2}-q_{2}q_{1}+q_{2}p_{1}\right)d=$$

$$\left(1-p_{2}\left(q_{1}+p_{1}\right)+p_{1}q_{2}-q_{2}q_{1}\right)d=$$

$$\left(q_{2}+p_{1}q_{2}-q_{2}q_{1}\right)d=q_{2}\left(1+p_{1}-q_{1}\right)d=q_{2}\left(1-q_{1}+p_{1}\right)d=2p_{1}q_{2}d$$

$$\delta_{b_{1}b_{2}}=-p_{1}a_{1}-p_{2}a_{2}-\left(p_{1}q_{2}+q_{1}p_{2}\right)d-\left(-p_{1}\alpha_{1}-p_{2}\alpha_{2}\right)=$$

$$-p_{1}a_{1}-p_{2}a_{2}-\left(p_{1}q_{2}+q_{1}p_{2}\right)d+p_{1}\left(a_{1}+\left(q_{2}-p_{2}\right)d\right)+p_{2}\left(a_{2}+\left(q_{1}-p_{1}\right)d\right)=$$

$$-\left(p_{1}q_{2}+q_{1}p_{2}\right)d+p_{1}\left(q_{2}-p_{2}\right)d+p_{2}\left(q_{1}-p_{1}\right)d=$$

$$-\left(p_{1}q_{2}+q_{1}p_{2}\right)d+\left(p_{1}q_{2}-p_{1}p_{2}+p_{2}q_{1}-p_{1}p_{2}\right)d=-2p_{1}p_{2}d$$

See Table A1 for more details.

**Table A1:** Values of genotypes in a two-allele system, measured as deviation from the population mean

Hybrid Genotypes	Frequency	Assigned genotypic values	Deviations from population mean $E\left(G\right)$		
			$G^{*}$	$g_{A^{\left(1\right)}}+g_{A^{\left(2\right)}}$	$g_{D}$
$B_{1}B_{2}$	$p_{1}p_{2}$	$a_{1}+a_{2}$	$q_{1}a_{1}+q_{2}a_{2}-\left(p_{1}q_{2}+q_{1}p_{2}\right)d$	$q_{1}\alpha_{1}+q_{2}\alpha_{2}$	$-2q_{1}q_{2}d$
$B_{1}b_{2}$	$p_{1}q_{2}$	$a_{1}+d$	$q_{1}a_{1}-p_{2}a_{2}+\left(1-p_{1}q_{2}-q_{1}p_{2}\right)d$	$q_{1}\alpha_{1}-p_{2}\alpha_{2}$	$2q_{1}p_{2}d$
$b_{1}B_{2}$	$q_{1}p_{2}$	$a_{2}+d$	$q_{2}a_{2}-p_{1}a_{1}+\left(1-p_{1}q_{2}-q_{1}p_{2}\right)d$	$-p_{1}\alpha_{1}+q_{2}\alpha_{2}$	$2p_{1}q_{2}d$
$b_{1}b_{2}$	$q_{1}q_{2}$	0	$-p_{1}a_{1}-p_{2}a_{2}-\left(p_{1}q_{2}+q_{1}p_{2}\right)d$	$-p_{1}\alpha_{1}-p_{2}\alpha_{2}$	$-2p_{1}p_{2}d$

$G^{*}$ is the total genotypic value of a hybrid deviated from the population mean.

$\left(g_{A^{\left(1\right)}}+g_{A^{\left(2\right)}}\right)$ is the additive-effect portion of a hybrid’s genotypic value.

$g_{D}$ is the dominance deviation of the hybrid.

### Properties of relationship matrices

Note that $G_{A^{\left(1\right)}}$ and $G_{A^{\left(2\right)}}$ have the following properties: the average value of the diagonal is 1, and the average value of the entire matrix is 0. For instance, the diagonal of $G_{A^{\left(1\right)}}$ sums to

$$\frac{\sum_{i}p_{1_{i}}{\left(1-p_{1_{i}}\right)}^{2}+q_{1_{i}}{\left(-p_{1_{i}}\right)}^{2}}{\sum_{i}p_{1_{i}}q_{1_{i}}}$$

which is equal to 1. In addition, the sum of the elements of $G_{A^{\left(1\right)}}$ is 0. Indeed, this sum can be written as

$$\frac{\sum_{i}\left(\begin{array}{cc} {p_{1}}_{i} & q_{1_{i}} \end{array}\right)\left(\;\begin{array}{c} 1-p_{1_{i}}\\ \;-p_{1_{i}} \end{array}\right){\left(\;\begin{array}{c} 1-p_{1_{i}}\\ \;-p_{1_{i}} \end{array}\right)}^{'}{\left(\begin{array}{cc} p_{1_{i}} & q_{1_{i}}\; \end{array}\right)}^{'}}{\sum_{i}p_{1_{i}}q_{1_{i}}}$$

which sums to 0. The same proof holds for $G_{A^{\left(2\right)}}$.

The diagonal of D sums to

$$\frac{\sum_{i}p_{1_{i}}p_{2_{i}}{\left(-2q_{1_{i}}q_{2_{i}}\right)}^{2}+p_{1_{i}}q_{2_{i}}{\left(2q_{1_{i}}p_{2_{i}}\right)}^{2}+q_{1_{i}}p_{2_{i}}{\left(2p_{1_{i}}q_{2_{i}}\right)}^{2}+q_{1_{i}}q_{2_{i}}{\left(-2p_{1_{i}}p_{2_{i}}\right)}^{2}}{\sum_{i}4p_{1_{i}}q_{1_{i}}p_{2_{i}}q_{2_{i}}}$$

which is equal to 1. In addition, the average value of the entire matrix D is 0. In effect, this sum can be written as

$$\frac{\sum_{i}\left(\begin{array}{cccc} p_{1_{i}}p_{2_{i}} & p_{1_{i}}q_{2_{i}} & q_{1_{i}}p_{2_{i}} & q_{1_{i}}q_{2_{i}} \end{array}\right)\left(\begin{array}{c} -2q_{1_{i}}q_{2_{i}}\\ 2q_{1_{i}}p_{2_{i}}\\ 2p_{1_{i}}q_{2_{i}}\\ -2p_{1_{i}}p_{2_{i}} \end{array}\right){\left(\begin{array}{c} -2q_{1_{i}}q_{2_{i}}\\ 2q_{1_{i}}p_{2_{i}}\\ 2p_{1_{i}}q_{2_{i}}\\ -2p_{1_{i}}p_{2_{i}} \end{array}\right)}^{'}\left(\begin{array}{cccc} p_{1_{i}}p_{2_{i}} & p_{1_{i}}q_{2_{i}} & q_{1_{i}}p_{2_{i}} & q_{1_{i}}q_{2_{i}} \end{array}\right)}{\sum_{i}4p_{1_{i}}q_{1_{i}}p_{2_{i}}q_{2_{i}}}$$

which sums to 0.

### Orthogonality

Next we prove orthogonality. In this model, $z_{1}$, $z_{2}$ and w are shifted to have mean zero for a population with these frequencies $\left(f=\left[p_{1}p_{2},\;p_{1}q_{2},\;q_{1}p_{2},\;q_{1}q_{2}\right]\right)$. Thus, the mean of additive value is zero because

$$\sum_{j}{z_{1}}_{j}\;f_{j}=\left(1-p_{1}\right)p_{1}p_{2}+\left(1-p_{1}\right)p_{1}q_{2}+\left(-p_{1}\right)q_{1}p_{2}+\left(-p_{1}\right)q_{1}q_{2}=0$$

$$\sum_{j}{z_{2}}_{j}\;f_{j}=\left(1-p_{2}\right)p_{1}p_{2}+\left(-p_{2}\right)p_{1}q_{2}+\left(1-p_{2}\right)q_{1}p_{2}+\left(-p_{2}\right)q_{1}q_{2}=0$$

and the mean of dominant deviations is also zero because

$$\sum_{j}w_{j}\;f_{j}=\left(-2q_{1}q_{2}\right)p_{1}p_{2}+\left(2q_{1}p_{2}\right)p_{1}q_{2}+\left(2p_{1}q_{2}\right)q_{1}p_{2}+\left(-2p_{1}p_{2}\right)q_{1}q_{2}=0$$

These equations correspond to the first requirement of orthogonality in Cockerham's (1954) model.

The second requirement can be expressed as $\sum_{i,j}f_{i,j}{z_{1}}_{i}z_{1_{j}}=0,\;\sum_{i,j}f_{i,j}{z_{2}}_{i}z_{2_{j}}=0$, $\sum_{i,j}f_{i,j}w_{i}w_{j}=0$, *e.g.* the contrasts have 0 mean across all possible pairs of genotypes (Cockerham 1954). For the first two, this can be written as $\sum_{i,j}f_{i,j}{z_{1}}_{i}z_{1_{j}}=\sum_{i,j}f_{i}z_{i}z_{j}f_{j}=\left(\begin{array}{cc} p_{1} & q_{1} \end{array}\right)\left(\begin{array}{c} 1-p_{1}\\ -p_{1} \end{array}\right){\left(\begin{array}{c} 1-p_{1}\\ -p_{1} \end{array}\right)}^{'}{\left(\begin{array}{cc} p_{1} & q_{1} \end{array}\right)}^{'}=0$ where $\left(\begin{array}{cc} p_{1} & q_{1} \end{array}\right)$ are frequencies of each genotype at the pure line and $\left(\begin{array}{c} 1-p_{1}\\ -p_{1} \end{array}\right)$ are the values of z for each genotype. Similarly, $\sum_{i,j}f_{i,j}w_{i}w_{j}=\sum_{i,j}f_{i}w_{i}w_{j}f_{j}=\left(\begin{array}{cccc} p_{1}p_{2} & p_{1}q_{2} & q_{1}p_{2} & q_{1}q_{2} \end{array}\right)\left(\begin{array}{c} -2q_{1}q_{2}\\ 2q_{1}p_{2}\\ 2p_{1}q_{2}\\ -2p_{1}p_{2} \end{array}\right){\left(\begin{array}{c} -2q_{1}q_{2}\\ 2q_{1}p_{2}\\ 2p_{1}q_{2}\\ -2p_{1}p_{2} \end{array}\right)}^{'}{\left(\begin{array}{cccc} p_{1}p_{2} & p_{1}q_{2} & q_{1}p_{2} & q_{1}q_{2} \end{array}\right)}^{'}=0$

Once the orthogonality of the one-locus formulation is proved, the orthogonal scales for the interactions in the multi-locus case can be generated by the Kronecker product. The extension of model using the Kronecker product guarantees the orthogonality of the multi-locus formulation (Van Loan 2000; Álvarez-Castro and Carlborg 2007).

## APPENDIX B

Variance components estimates for GCA-models excluding “residual genetic” r effects and broad-sense heritabilities are shown in Table A2.

**Table A2:** Estimated posterior means and standard deviation (in parenthesis) of genetic variance component obtained with GCA-model without including residual genetic effects from Dent and Flint groups

Model Code	Additive		Dominance	Epistasis				
	$\sigma_{A^{\left(1\right)}}^{2},\sigma_{A^{\left(2\right)}}^{2}$ or $\sigma_{A^{\left(H\right)}}^{2}$		$\sigma_{D}^{2}$ or $\sigma_{D^{\left(H\right)}}^{2}$	$\sigma_{AA^{\left(1,1\right)}}^{2}$	$\sigma_{AA^{\left(2,2\right)}}^{2}$	$\sigma_{AA^{\left(1,2\right)}}^{2}$ or $\sigma_{AA^{\left(H\right)}}^{2}$	$\sigma_{e}^{2}$	$H^{2}$
GCA:A	33.89 (5.52)	23.35 (4.56)					18.01 (0.79)	0.76 (0.02)
GCA:AD	31.89 (5.20)	22.56 (4.46)	4.38 (0.77)				15.03 (0.80)	0.80 (0.02)
$\mathrm{GCA}:A{\left(AA\right)}^{\left(1,2\right)}$	31.38 (5.13)	22.53 (4.42)				5.58 (0.96)	13.68 (0.85)	0.81 (0.02)
$\mathrm{GCA}:AD{\left(AA\right)}^{\left(1,2\right)}$	31.08 (5.06)	22.11 (4.42)	2.97 (0.58)			4.20 (0.87)	13.36 (0.81)	0.82 (0.02)
$\mathrm{GCA}:AD{\left(AA\right)}^{\left(1,1\right)}{\left(AA\right)}^{\left(2,2\right)}{\left(AA\right)}^{\left(1,2\right)}$	22.30 (5.20)	14.42 (4.11)	2.55 (0.56)	7.19 (2.50)	8.06 (2.89)	3.63 (0.82)	13.53 (0.81)	0.81 (0.02)

GCA-model is a model that successively added additive effects (A), dominance effects (AD), and additive-by-additive genetic effects (AD(AA)). The additive-by-additive epistatic effects can be interactions between loci within group (${\left(AA\right)}^{\left(11\right)}$ and ${\left(AA\right)}^{\left(22\right)}$), across groups ${\left(AA\right)}^{\left(12\right)}$ or within hybrids ${\left(AA\right)}^{\left(H\right)}$. Superscripts 1 and 2 in parenthesis refers to Dent and Flint heterotic groups, respectively.

In GCA-model, the variances are: additive ($\sigma_{A^{\left(1\right)}}^{2}$ and $\sigma_{A^{\left(2\right)}}^{2}$), dominance ($\sigma_{D}^{2}$), and additive-by-additive epistasis within groups ($\sigma_{AA^{\left(1,1\right)}}^{2}$ and $\sigma_{AA^{\left(2,2\right)}}^{2}$) and additive-by-additive epistasis between groups ($\sigma_{AA^{\left(1,2\right)}}^{2}$). $\sigma_{e}^{2}$ is the residual variance and $H^{2}$ is the genomic broad-sense heritability.
